# Systems Biology Analysis of Gene Expression during *In Vivo Mycobacterium avium paratuberculosis* Enteric Colonization Reveals Role for Immune Tolerance

**DOI:** 10.1371/journal.pone.0042127

**Published:** 2012-08-17

**Authors:** Sangeeta Khare, Sara D. Lawhon, Kenneth L. Drake, Jairo E. S. Nunes, Josely F. Figueiredo, Carlos A. Rossetti, Tamara Gull, Robin E. Everts, Harris A. Lewin, Cristi L. Galindo, Harold R. Garner, Leslie Garry Adams

**Affiliations:** 1 Department of Veterinary Pathobiology, College of Veterinary Medicine and Biomedical Sciences, Texas A&M University, College Station, Texas, United States of America; 2 Seralogix, Limited Liability Company, Austin, Texas, United States of America; 3 Department of Animal Sciences, University of Illinois at Urbana-Champaign, Urbana, Illinois, United States of America; 4 Eugene McDermott Center for Human Growth and Development, University of Texas Southwestern Medical School, Dallas, Texas, United States of America; University of Hyderabad, India

## Abstract

Survival and persistence of *Mycobacterium avium* subsp. *paratuberculosis* (MAP) in the intestinal mucosa is associated with host immune tolerance. However, the initial events during MAP interaction with its host that lead to pathogen survival, granulomatous inflammation, and clinical disease progression are poorly defined. We hypothesize that immune tolerance is initiated upon initial contact of MAP with the intestinal Peyer's patch. To test our hypothesis, ligated ileal loops in neonatal calves were infected with MAP. Intestinal tissue RNAs were collected (0.5, 1, 2, 4, 8 and 12 hrs post-infection), processed, and hybridized to bovine gene expression microarrays. By comparing the gene transcription responses of calves infected with the MAP, informative complex patterns of expression were clearly visible. To interpret these complex data, changes in the gene expression were further analyzed by dynamic Bayesian analysis, and genes were grouped into the specific pathways and gene ontology categories to create a holistic model. This model revealed three different phases of responses: i) early (30 min and 1 hr post-infection), ii) intermediate (2, 4 and 8 hrs post-infection), and iii) late (12 hrs post-infection). We describe here the data that include expression profiles for perturbed pathways, as well as, mechanistic genes (genes predicted to have regulatory influence) that are associated with immune tolerance. In the Early Phase of MAP infection, multiple pathways were initiated in response to MAP invasion via receptor mediated endocytosis and changes in intestinal permeability. During the Intermediate Phase, perturbed pathways involved the inflammatory responses, cytokine-cytokine receptor interaction, and cell-cell signaling. During the Late Phase of infection, gene responses associated with immune tolerance were initiated at the level of T-cell signaling. Our study provides evidence that MAP infection resulted in differentially regulated genes, perturbed pathways and specifically modified mechanistic genes contributing to the colonization of Peyer's patch.

## Introduction

*Mycobacterium avium* subsp. *paratuberculosis* (MAP) causes a chronic enteric infection (Johne's disease) in cattle and other ruminants that is established after ingestion of bacteria followed by invasion and colonization of the intestinal mucosa. The major hurdle in understanding MAP infection is its chronic nature and delayed onset of clinical symptoms. Much is known regarding the host response of chronically infected cattle, but the understanding of the early events in the host is limited. In the jejunal-ileal Peyer's patches, MAP gain entry in intestinal mucosa via interaction with M cells, goblet cells, epithelial cells, dendritic cells or macrophages [1]–[3]. Lesions that are characterized by aggregates of macrophages, epithelioid cells, and giant cells develop in the intestinal mucosa of experimentally infected neonatal calves within 5 months [4]. Moreover, systemic humoral and cellular immune responses develop within months after instillation of MAP into the tonsillar crypts of neonatal calves [5]. Mononuclear cells isolated from the intestine of cows subclinically infected with MAP showed a state of tolerance [6], [7]. Several studies have focused on the role of circulating mononuclear phagocytes during MAP infection [8]–[12]. It appears that mucosal immunological tolerance is required for persistent infection. However, the outcome of the host-pathogen interaction depends on the collective response of various cell types that are present at the mucosal surface. Additionally, knowledge of which components of the host response are involved in the activation of innate immunity is poorly understood in chronic infections. Thus, more comprehensive knowledge is needed regarding the pathogen interaction within the host milieu of the natural site of infection. Toward this goal, we have successfully established the perinatal calf ligated ileal loop model for studying early changes in the mucosa during MAP infection [2].

About 65% of U.S. herds are infected with MAP [13]. This level of infection definitely leads to contamination of the environment via contaminated water supply, dust bio-aerosol, milk and food supply. This contaminated environment not only affects the spread of MAP among animals, it may also be associated with the human intestinal disorder, Crohn's disease [14]–[18]. Interestingly, MAP has been isolated from intestinal tissue, as well as, peripheral blood of human patients suffering with a similar granulomatous inflammatory disease known as Crohn's disease [19]. Several pathogens, including MAP, have been claimed to be associated with the Crohn's disease in human [20], [21]. Like Johne's disease, Crohn's disease also affects a pediatric population. In a recent study, identification of MAP in gut tissue and blood from pediatric inflammatory bowel disease patients suggests the possible involvement of MAP in the early stages of development of Crohn's disease in children [22]. Crohn's disease is likely to be more than one disease, which complicates research. Furthermore, microbial contact or invasion may be confined to parts of the alimentary tract that are relatively inaccessible to tissue sampling, such as the ileum or jejunum. The *in vivo* perinatal calf jejunal-ileal loop model provides an ideal animal model to more precisely study the early pathogenesis of MAP or other microbial pathogens in Crohn's disease.

In the present study, we hypothesized that MAP induces an immune tolerance in Peyer's patch very soon after invasion. To test our hypothesis, we analyzed the temporal gene expression in detail during the early stages of colonization of Peyer's patch by MAP. We applied a systems biology approach to analyze the complex microarray data enabling us to identify specific cellular pathway perturbations and predicted cell type involvement during the early infection and colonization. Furthermore, we identified innate response signatures (mechanistic genes) adequate to envisage the subsequent adaptive immune responses leading to persistent MAP infection. The aims of this study were two fold, 1) to utilize a systems biology analytical approach to gain new insights regarding the most perturbed cellular pathways; and 2) to provide evidence for tolerance related components of mucosal immunity during early MAP infection.

## Materials and Methods

### Culture of MAP

*Mycobacterium avium* subsp. *paratuberculosis* (ATCC 19698) from American Type Culture Collection (ATCC), Manassas, VA, was grown aerobically in 7H9 broth (Difco Laboratories, Detroit, MI) supplemented with 2.5% (vol/vol) glycerol (Sigma Chemical Co., St. Louis, MO), oleic acid-albumin-dextrose-catalase (Difco Laboratories, Detroit, MI), 0.05% Tween 80 (Sigma Chemical Co., MO), and 2 mg/liter of Mycobactin J (Allied Monitor, Inc., Fayette, MO). Single-cell suspensions and enumeration of MAP were done as described earlier [23].

### Animals

Four clinically healthy male, unrelated Holstein-Friesian calves, 3–4 weeks of age and weighing 45–55 kg, were used in the experiment under an approved animal use protocol in accordance with animal use policy under the supervision of the Texas A & M University Institutional Animal Care and Research Advisory Committee (AUP 2007-70). The calves were fed antibiotic-free milk replacer twice daily and water *ad libitum*. All of the calves were tested for the presence of *Salmonella* spp. and MAP. Fecal specimens and rectal swabs were collected from calves two weeks prior and immediately before the experiment. Fecal specimens were prepared for the PCR for detection of MAP [23] and culture based detection of *Salmonella* [24]. Only calves with negative tests for these pathogens were used in these experiments.

### Bovine Ligated Jejunal-Ileal Loop Surgery

The calves were fasted for 24 hrs prior to the non-survival surgery, anesthetized and maintained analgesic for the course of the 12 hrs experiment. In brief, anesthesia was induced with Propofol (Abbot Laboratories, Chicago, IL) followed by placement of an endotracheal tube and maintenance with isoflurane (Abbot Laboratories, Chicago, IL) for the duration of the experiment. The detailed bovine ligated ileal loop surgery procedure is described elsewhere [2]. Loops were prepared exclusively in the 1.0–1.2 meter long Peyer's patch that is proximal to the ileocecal junction and included jejunum and ileum. Loops were inoculated with 3.0 ml of PBS or 3×10^9^ cfu of MAP in 3.0 ml of PBS. At 0.5, 1, 2, 4, 8 and 12 hrs after inoculation, one each of control (PBS inoculated) and experimental (MAP inoculated) loops were excised. Samples for bacteriologic culture and RNA extraction were collected as described below. Throughout the experimental procedure, the calves were monitored for vital signs (blood pressure, heart rate, hydration status, anesthesia depth and temperature). The calves were euthanized with a rapid overdose (single bolus at 60 mg/lb IV) of pentobarbital sodium after the final samples were collected at 12 hrs post-inoculation.

### Bacteriology

A 6 mm biopsy punch was used to collect two intestinal mucosal samples from each loop of Peyer's patch for bacteriology. Intestinal biopsy samples were washed three times in PBS, weighed, placed in the Whirl-Pak™ bag, homogenized in a Colworth-Stomacher blender in PBS, and serially diluted. The tissue extracts were plated onto Herrold egg yolk media containing amphotericin, nalidixic acid, vancomycin (Becton Dickinson and Company, Sparks, MD) with or without Mycobactin J and incubated at 37°C. The cultures were observed visually weekly for any contamination, and the final counts of colony forming units were recorded on week 16.

### Extraction and Quality Analysis of RNA

A 6 mm biopsy punch was used to collect 8 intestinal mucosal samples (at each time) of Peyer's patch from the excised PBS control and MAP infected loops for the extraction of RNA at 0.5 1, 2, 4, 8, and 12 hrs post-infection. The tissue was immediately minced with a scalpel blade and transferred to TRI Reagent™ (Molecular Research Center, Cincinnati, OH). Two biopsy punches (approximately 0.1 mg of tissue) were placed into 0.5 ml of TRI reagent™. Tissues were further disrupted with hand-held mechanical tissue grinder equipped with a RNase, DNase free plastic disposable pestle. The RNA extraction was done using the recommended protocol from the manufacturer (Molecular Research Center, Cincinnati, OH). The resultant RNA pellet was re-suspended in DEPC-treated water (Ambion, Austin, TX). Genomic DNA was removed by RNase-free DNase I treatment (DNA-free, Ambion) according to the manufacturer's instructions, and samples were stored at −80°C until used. RNA concentration was quantified by measuring absorbance at λ260 nm using a NanoDrop® ND-1000 (NanoDrop, Wilmington, DW). RNA quality was evaluated by measuring ratio of absorbance at λ260 nm to absorbance at λ280 nm, agarose gel electrophoresis, and using a Nano-Chip® on an Agilent 2100 Bioanalyzer (Agilent, Palo Alto, CA). All the RNA samples used in this study were of good to excellent quality (results not shown), and had distinct 18S and 28S rRNA peaks and RNA size distribution. Ten µg of this high quality experimental RNA was reverse transcribed and used to create indirectly labeled Cy5 cDNA as starting material for each microarray.

### Bovine Reference RNA

Bovine reference RNA was prepared in-house and constituted of equal proportions of total RNA from Madin-Darby bovine kidney and bovine B lymphocyte cell lines, and fresh bovine brain cortex and cerebellum [24], [25]. This reference RNA has been shown to hybridize to the great majority of the open reading frames (ORFs) represented on the microarray. The reference RNA was treated in the same way as the experimental and control RNA for the co-hybridization with each sample on the microarray.

### Bovine Microarrays, Sample Preparation and Hybridization

Bovine microarrays were obtained from the W. M. Keck Center (University of Illinois at Urbana-Champaign). These custom microarrays consisted of 70-mer oligonucleotides representing 13,258 unique oligos with 12,220 cattle ORFs. A detailed description of the design and development of microarray has been published elsewhere [26]. Labeling of cDNA and hybridization to microarray have been described previously [27]. Briefly, 10 µg of RNA was reverse transcribed using Superscript III reverse transcriptase (Invitrogen, Carlsbad, CA) and labeled with amino-allyl-UTP (Ambion, Austin, TX). Cy3 and Cy5 dye esters were covalently linked to the amino-allyl group by incubating the samples with the dye esters in 0.1 M sodium carbonate buffer. cDNA from bovine experimental samples (i.e. from MAP infected and PBS control loops) were labeled with Cy5 and co-hybridized against Cy3 labeled cDNA generated from the bovine reference RNA sample 13K bovine 70-mer oligonucleotides array. Prior to hybridization, the microarrays were denatured by exposing to steam from boiling water for three seconds, cross-linked ultraviolet light and then immersed in pre-hybridization buffer (5× sodium chloride, sodium citrate buffer (SSC), 0.1% sodium dodecyl sulfate (SDS) (Ambion, Austin, TX), 1% bovine serum albumin (BSA) at 42°C for a minimum of 45 min followed by four washes in RNase-, DNase-free, distilled water, immersion in 100% isopropanol for 10 seconds, and dried by centrifugation. Slides were hybridized at 42°C for approximately 40 hrs in a dark humid chamber (Corning, Corning, NY) and washed for 10 min at 42°C with low stringency buffer (1×SSC, 0.2% SDS), followed by two 5 min washes in a higher stringency buffer (0.1×SSC, 0.2% SDS and 0.1×SSC) at room temperature in the dark with mild agitation.

### Data Acquisition and Normalization of Hybridized Spots on Microarrays

Slides were scanned using a GenePix 4100 laser scanner (Axon Instruments Inc., Foster City, CA). The spots with fluorescent signal representing genes on the arrays were adjusted for background and normalized to internal controls using image analysis software. If the fluorescent signal of any spot was below the background, they were disregarded in all analyses. An average of 56% of spots in experimental samples (Cy5 channel; laser excitation = 635), 77% of spots in reference samples (Cy3 channel; laser excitation = 532), and 54% of spots in the combined samples (Cy5/Cy3 channels) had a signal to noise ratio greater than three.

### Microarray Data Analysis

Microarray data analysis was performed by two different methods. In the first method, arrays were normalized by scaling against the average reference intensity value (i.e., average across all microarrays), normalized by the global mean, and then log transformed before statistical analyses were performed. Signals flagged as “non-acceptable" by GenePix (Axon Instruments Inc. Foster City, CA) were removed across all arrays in order to ensure that subsequent analyses for each time point were comparable. Pairwise comparisons of averaged signal values and Student's *t* test were performed using GeneSifter software (VizX Labs, Seattle, WA). Normalization of each sample was performed against the bovine reference RNA signals across slides and within each slide (across the duplicate spots). Before normalization, duplicate spots were separated and treated as technical replicates. The two spots representing a single gene were therefore required to “agree," based on subsequent analysis steps. A fold-change of at least 1.5-fold and *P*<0.05 was expected for a difference in signal to be considered statistically significant. All possible individual pairwise comparisons between controls and infections were also performed using Spotfire DecisionSite software (Spotfire, Inc., Somerville, MA). Genes were further filtered using these various comparisons in order to ensure biological relevance (i.e., that observed differences were not the result of random variation between uninfected animals) and consistency (i.e., reproducibility across experiments).

In the second method, more recent computational tools termed the BioSignature Discovery System (BioSignatureDS) (Seralogix, LLC, Austin, TX) were employed to conduct comparative pathogenicity analysis and modeling. This approach for genomic data analysis and modeling at the system biology level offers an integrated view of biological mechanisms and networks of interactions. Specifically for the analysis reported herein, the tools were used to: 1) determine significant gene modulations via a *z*-score sliding window threshold technique and fold change; 2) conduct biological system level analysis employing Bayesian network models for scoring and ranking of metabolic pathways, signaling pathways and gene ontology (GO) groups; 3) conduct Bayesian candidate mechanistic gene analysis to identify genes within the network models that are most responsible for causing pathway and GO group perturbations; and 4) create a genetic network system model derived from the candidate mechanistic genes and their genetic interactions. More detailed description of the computational techniques employed by BioSignatureDS was described in our previous publication [24].

### Microarray Data Deposited in the Gene Expression Omnibus

The microarray data were deposited in the Gene Expression Omnibus at the National Center for Biotechnology Information (http://www.ncbi.nlm.nih.gov/geo/) Accession **#** GSE13888.

### Induction of RNAi in HeLa Cells

HeLa cells (ATCC CCL-2.2, from ATCC, Manassas, VA) were cultured in F12K medium supplemented with L-glutamine and 10% heat inactivated fetal bovine serum (HI-FBS). On day 1, approximately 4×10^4^ HeLa cells were placed into 24 well cell culture Plates (Corning, Corning, NY) in 0.4 ml of culture medium and placed in a 37°C incubator with 5% CO_2_. The following day, for each 24-well culture to be transfected, 50 µl of serum-free cell growth medium (OPTI-MEM, Invitrogen, Carlsbad, CA) was mixed in separate compartments with *Silencer*® validated siRNAs (Ambion, Austin, TX). The siRNAs were used at final concentrations according to the manufacturer's protocol: mitogen-activated protein kinase 1 (MAPK1) (ID 1449) 30 nM, MAPK1 (ID 1544) 100 nM, epidermal growth factor EGF (ID 645) 50 nM, and negative control siRNA (AM4635) 50 nM. Simultaneously, 1 µl of TransFectin® lipid reagent (Bio-Rad, Hercules, CA) was diluted into 50 µl of serum-free cell growth medium for each 24-well culture to be transfected. The diluted siRNA was combined and mixed with the diluted TransFectin® reagent. Additional negative controls consisted of cells to which either 100 µl of OPTI-MEM or 100 µl of OPTI-MEM with 1 µl of TransFectin® were added. After 20 min incubation at RT, 100 µl of the siRNA-TransFectin mixture were added to the 400 µl of F12K cell culture media on the cells. On day 3, one ml of F12K medium with 10% FBS was added to the each well. On day 4, transfected cells were infected with MAP at a multiplicity of infection (MOI) of 10 bacteria per HeLa cell, and bacterial invasion was determined as described below. For validation of RNAi efficiency, RNA from transfected cells was extracted at the same time of infection (i.e. 48 hrs post-transfection) using Tri Reagent (MRC, Cincinnati, OH) according to the manufacturer's protocol. Contaminant genomic DNA was removed by RNase-free DNase I treatment (Ambion) according to the manufacturer's instructions, and samples were stored at −80°C until used. RNA concentration was quantitated by NanoDrop® ND-1000 (NanoDrop, Wilmington, DW). Target mRNA levels were measured by qRT-PCR as previously described for microarray validation [24].

### Functional Assessment of siRNA Transfected HeLa Cells For Invasion of MAP

HeLa cells were cultured as described above. Before adding MAP, the HeLa cells from 2 wells were detached and counted. Invasion assays were performed by removing 1.2 ml of the medium overlying the HeLa cells monolayers and adding 100 µl of a bacterial inoculum re-suspended in cell culture media, at a MOI of 10∶1. Bacteria were centrifuged onto the cells at 800×*g* for 2 min followed by 1 hr of incubation at 37°C. Then, cells were washed three times with PBS to remove extracellular bacteria and re-incubated with F12K media supplemented with 100 µg ml^−1^ of gentamicin solution (Sigma, St. Louis, MO) for 2 hrs. After antibiotic treatment, infected cultures were washed three times with PBS and then lysed with 0.1% Triton X-100 (Sigma). Lysates were serially diluted and cultured on Herrold egg yolk media supplemented with Mycobactin J and amphotericin, nalidixic acid, and vancomycin for quantification of colony-forming units (CFU). Duplicate wells were used for each experiment, and the experiments were performed three times.

### Kinetics of Trans-Epithelial Resistance of Polarized Epithelial Cells During Invasion of pathogen

T84 cells (ATCC 14028, from ATCC, Manassas, VA) were grown in DMEM/F12 medium (Gibco, Life Technologies) containing 1.2 g of sodium bicarbonate per liter, 2.5 mM L-glutamine, 15 mM HEPES, and 0.5 mM sodium pyruvate (Gibco, Life Technologies) supplemented with 10% fetal calf serum. T84 cells were polarized by seeding 4×10^5^cells/well on the apical compartment of 12-mm-diameter Transwell plates (polycarbonate membrane with a pore size of 0.4 µm; Corning Costar) and 1.5 ml of media was added to the basolateral compartment. The medium was changed every other day, and the transepithelial electrical resistance (TER) was measured after 7–8 days. After the cells reached a TER of at least1,500 µ/cm^2^, they were incubated overnight in fresh medium, and the invasion assay was performed on the following day using a MOI of 10∶1. The change in the TER was measured at various time intervals, starting at 2 mins post-infection to 24 hrs post-infection, with a voltmeter (Millipore-ERS resistance meter; Millipore, Bedford, Mass.).

### Statistical Analysis of Bacteriology

Colonization of tissue samples and transfected HeLa cells was considered to be positive when MAP was detected by bacteriological culture. Tissue burden was defined as the number of colony forming units per milligram of tissue. The statistical significance of differences was calculated using two-tailed Student's *t* test.

## Results

### Invasion of Ileal Mucosa by MAP

MAP was recovered from the MAP-inoculated ileal tissues at all the time points post-infection (data not shown). No bacteria were detected in the PBS inoculated loops. Among the infected loops, no significant changes in the number of MAP were detected at any times post-inoculation (0.5–12 hrs).

### Bovine Peyer's Patches Inoculated with MAP Reveals a Complex Temporal Pattern of Transcriptional Profile

In order to gain detailed insight into the changes in the transcriptional profile of genes in bovine intestinal Peyer's patch mucosa inoculated with 3×10^9^ cfu of MAP (30, 60, 120, 240, 480, and 720 min post-infection), initially the microarray data analysis was performed by using GeneSifter software (where a fold-change of at least 1.5-fold and *P*<0.05 was required for a difference in signal to be considered statistically significant, Table 1). Classical analysis of the altered gene expression by GeneSifter, provides the static changes in the experimental conditions with a profound spectrum of data; however, after filtering the data into the biological relevant and significant genes, a limited number of genes were found to have statistically significant expression.

**Table 1:** Classical Analysis of Microarray by GeneSifter Software.

Classical Analysis by GeneSifter	MAP vs PBS		
	Upregulated	Downregulated	Total
**30 Min**			
Average Fold Change ≥1.5-fold	519	2,468	2,987
Statistically significant (p value≤0.05)	9	202	211
Biologically relevant and consistent	0	25	25
**60 min**			
Average Fold Change ≥1.5-fold	644	2,185	2,829
Statistically significant (p value≤0.05)	74	183	257
Biologically relevant and consistent	1	49	50
**120 min**			
Average Fold Change ≥1.5-fold	888	1,987	2,875
Statistically significant (p value≤0.05)	99	168	267
Biologically relevant and consistent	15	32	47
**240 min**			
Average Fold Change ≥1.5-fold	688	1,585	2,273
Statistically significant (p value≤0.05)	16	199	215
Biologically relevant and consistent	2	42	44
**480 min**			
Average Fold Change ≥1.5-fold	1,745	727	2,472
Statistically significant (p value≤0.05)	215	35	250
Biologically relevant and consistent	27	5	32
**720 min**			
Average Fold Change ≥1.5-fold	2,030	2,278	4,308
Statistically significant (p value≤0.05)	83	35	118
Biologically relevant and consistent	7	6	13

As shown in Table 1, no genes were significantly up-regulated in MAP-infected animals at the earliest time point tested (30 min), and only modest numbers of genes (less than 30 by 8 hr) were increased in expression over the experimental time course. In contrast, at the earliest time points, MAP-infected loops had down-regulation in gene expression. This observation clearly reflected that measurement of subtle changes may be undetected if: 1) the quantity of the change is very small to measure, or 2) if experiments are conducted using out bred populations, or 3) if the sample used is a heterogeneous population of several cell types expressing different levels of expression of same gene. To overcome these challenges, we extended the analysis to create a temporal relationship of various genes using BiosignatureDS tools that employed a method termed the Dynamic Bayesian Gene Group Activation (DBGGA)(Seralogix, Austin, TX). Bayesian network models were created for all the known signaling and metabolic pathways and for Gene Ontology (GO) biological processes. The models were trained with the control group data (uninfected) with the experimental data (infected) used as evidence to test how different experimental data are applied in fitting the control model. This difference is determined by measuring the negative log-likelihood that, in turn, was transformed to a *z*-score test statistics that is referred to, here after, as the Bayesian *z*-score. This method ranked groups of genes at each time point and across all time points to determine differences between experimental conditions [24], [28]. Similarly, how well the results of individual genes fit a model was also determined, producing Bayesian *z*-scores for each gene within a pathway or GO category. This method used a less stringent spot quality filtering technique; a more sophisticated universal reference normalization method in conjunction with Lowess correction; and a Bayesian variance estimator that infers a better prediction of the standard deviation for genes which have a low number of biological replicates [29], [30]. Using this approach, the contribution of small changes in key regulatory genes was taken into account.

### Classification of Perturbed Pathways using Bayesian *z*-scoring Divides the Host Transcriptional Response into Three Phases

As indicated by the host system level pathway analyses that identified significantly perturbed pathways over the experimental time course, there were three logical classification phases (Early, Intermediate and Late) in which it is proposed that invasion occurs in the Early Phase and longer term evasion and host immune tolerance occurs in all three phases. The Early Phase of infection consisted of the 30 and 60 min time periods post-infection. The Intermediate Phase consisted of the 120, 240, and 480 min time periods post-infection, and the Late Phase infection at 720 minutes post-infection. A comprehensive Bayesian *z-*score list of significantly perturbed pathways identified for MAP inoculated loop, passing the 97.5% confidence threshold is provided in Tables 2, 3, and 4, organized into Early, Intermediate, and Late Phase responses, respectively. The rank order of pathways in Tables 2, 3 and 4 are based on the Bayesian *z-*scores ranging from largest to smallest for the first time point in each of the phases (i.e., t = 30 minutes for Early, t = 120 minutes for Intermediate, and t = 720 minutes for Late). These tables organize the pathways in terms of their state of activation or suppression. Of 220 pathways scored by the DBGGA method, the Early Phase response had 82 significantly perturbed signaling and metabolic pathways, the Intermediate Phase had 70, and the Late Phase had 117. There were 30 pathways that were significantly perturbed in common to all three phases and are highlighted in *italics* in Tables 2, 3 and 4. The Early Phase had 23 pathways that were uniquely perturbed compared to the other phases while the Intermediate Phase had only 11, and the Late Phase had 45 uniquely perturbed pathways. These uniquely perturbed pathways are indicated in these tables with an “*" before the name of the pathway. As a supplement to Tables 2, 3 and 4, a heat map of all pathway scores is provided in Figure S1 to better visualize the temporal patterns and the degree of perturbation at each time point post infection.

**Table 2:** Early Phase Significantly Perturbed Pathways Bayesian *z*-Scores.

Activated Pathways	t = 30	t = 60	Activated Pathways (con't)	t = 30	t = 60	Suppressed Pathways	t = 30	t = 60
Parkinson's disease	4.61	−0.31	C21-Steroid hormone metabolism	2.84	−0.06	Peptidoglycan biosynthesis	−1.55	−2.65
Porphyrin and chlorophyll metabolism	4.35	0	*Tyrosine metabolism*	2.84	−0.23	Lysine biosynthesis	−2.28	0
*Complement and coagulation cascades*	3.77	−1	*PPAR signaling pathway*	2.82	−0.81	*Tight junction	−2.28	−0.77
Wnt signaling pathway	3.59	0.9	*Androgen and estrogen metabolism	2.79	−0.57	Thyroid cancer	−2.36	−1.33
*Histidine metabolism	3.57	−0.38	Lysine degradation	2.77	−0.49	*Trefoil Factors Initiate Mucosal Healing	−2.36	0.23
*Pyruvate metabolism*	3.49	0.04	*Bile acid biosynthesis*	2.76	−0.1	*Cell Communication*	−2.37	−1.21
Olfactory transduction	3.39	0	*Glycosphingolipid biosynthesis - globoseries	2.74	1.34	*Endometrial cancer*	−2.45	1.82
*Tetrachloroethene degradation*	3.32	0.88	*Acute myeloid leukemia*	2.73	−1.07	*Pentose phosphate pathway	−2.46	−2.06
*Hematopoietic cell lineage*	3.32	−0.42	Fructose and mannose metabolism	2.71	−2.96	C5-Branched dibasic acid metabolism	−2.55	0
*CD40L Signaling Pathway*	3.28	0	*Aminophosphonate metabolism	2.7	0	DNA polymerase	−2.56	0.03
*microtubule-associated protein 1A*	3.27	−0.1	GnRH signaling pathway	2.7	−0.44	Anthrax Toxin Mechanism of Action pathway	−2.57	0
*Phosphatidylinositol signaling system	3.26	−0.2	*Caprolactam degradation	2.66	0	*Basal cell carcinoma*	−2.58	−1.26
Gap junction	3.23	−0.91	*Adipocytokine signaling pathway*	2.54	0	Fatty acid elongation in mitochondria	−2.62	0.67
Propanoate metabolism	3.22	0	*Urea cycle and metabolism of amino groups	2.54	0	Axon guidance	−2.64	−0.92
*Starch and sucrose metabolism*	3.17	−0.42	*Toll-like receptor signaling pathway*	2.51	−1.26	Reductive carboxylate cycle (CO2 fixation)	−3.02	0.82
*Cytokine-cytokine receptor interaction*	3.16	−0.6	*Nicotinate and nicotinamide metabolism	2.49	−0.83	*Glyoxylate and dicarboxylate metabolism	−3.14	1.67
Retinol metabolism	3.16	−0.57	*Pentose and glucuronate interconversions*	2.48	−0.48	*Pathogenic Escherichia coli infection*	−3.15	0.01
*Folate biosynthesis*	3.14	0.8	*beta-Alanine metabolism	2.44	0	*One carbon pool by folate*	−3.18	1.99
*Tryptophan metabolism	3.12	1.31	*Lectin Induced Complement Pathway	2.43	−0.31	*Long-term potentiation*	−3.29	1.24
*Glycine, serine and threonine metabolism*	3.05	−0.56	*Butanoate metabolism*	2.42	0.65	*Citrate cycle (TCA cycle)*	−3.3	0.59
*Huntington's disease	3.04	−0.16	*Glycolysis/Gluconeogenesis*	2.4	−0.78	*Melanogenesis*	−3.49	−0.97
Inositol phosphate metabolism	3.02	0	Arachidonic acid metabolism	2.39	−1.5	*Long-term depression	−3.51	−0.22
*Biosynthesis of unsaturated fatty acids	2.97	1.54	Purine metabolism	2.37	0.26	*CCR3 signaling in Eosinophils pathway	−3.51	0.96
*Selenoamino acid metabolism	2.93	−1.64	Methionine metabolism	2.35	−1.2	Oxidative phosphorylation	−3.57	−0.98
Metabolism of xenobiotics by cytochrome P450	2.91	−1.07	Ether lipid metabolism	2.33	0	Fatty acid biosynthesis	−4.02	1.76
*Neuroactive ligand-receptor interaction*	2.87	−1.27	Arginine and proline metabolism	2.33	−0.1			
*Calcium signaling pathway*	2.87	−0.11	*Ascorbate and aldarate metabolism	2.33	−0.97			
*Glycosylphosphatidylinositol(GPI)-anchor biosynthesis*	2.85	0	*Glycerophospholipid metabolism	2.29	0			

**Table 3:** Intermediate Phase Significantly Perturbed Pathways Bayesian *z*-Scores.

Activated Pathways	t = 120	t = 240	t = 480	Activated Pathways (con't)	t = 120	t = 240	t = 480	Suppressed Pathways	t = 120	t = 240	t = 480
C21-Steroid hormone metabolism	2.77	2.06	1.26	*PPAR signaling pathway*	0.91	1.82	3.58	ECM-receptor interaction	−0.08	2.53	2.63
*Sulfur metabolism	2.62	0.69	−1.82	ErbB signaling pathway	0.88	2.42	−1.65	*3-Chloroacrylic acid degradation	−0.18	−2.44	2.86
Chondroitin sulfate biosynthesis	2.29	−1.15	2.29	*microtubule-associated protein 1A*	0.77	1.85	2.66	Carbon fixation	−0.19	−0.75	3.42
*CD40L Signaling Pathway*	2.23	−1.15	−2.46	*Pentose and glucuronate interconversions*	0.77	1.68	2.7	*Basal cell carcinoma*	−0.23	2.95	1.27
*Pyruvate metabolism*	1.95	2.24	2.68	*Cell Communication*	0.47	2.49	3.07	*Glycolysis/Gluconeogenesis*	−0.29	−1.96	3.08
*Bile acid biosynthesis*	1.9	3.12	3.17	Type I diabetes mellitus	0.12	−1.25	2.81	*Fluorene degradation	−0.39	−2.8	−0.77
*Hematopoietic cell lineage*	1.81	3.04	−2.01	*Fatty acid metabolism	0.04	2.09	2.53	*Pathogenic Escherichia coli infection*	−0.55	2.27	2.16
Stilbene, coumarine and lignin biosynthesis	1.8	−2.54	2.33	Small cell lung cancer	0	2.46	−1.67	Fatty acid elongation in mitochondria	−0.57	−2.53	−1.1
Lysine biosynthesis	1.75	2.36	0.18	*1,4-Dichlorobenzene degradation	0	2.24	1.61	*Acute myeloid leukemia*	−0.64	−1.8	−2.88
Cytokine Inflammatory Response	1.74	3.75	1.39	*Fluorobenzoate degradation	0	2.24	1.61	Inositol metabolism	−0.68	−2.41	0
*Adipocytokine signaling pathway*	1.72	2.12	2.69	RNA polymerase	0	1.62	2.24	Riboflavin metabolism	−0.75	1.61	3.3
*Complement and coagulation cascades*	1.41	2.63	3.01	*Heparan sulfate biosynthesis	0	1.55	2.32	*Tetrachloroethene degradation*	−0.79	3.3	2.39
Regulation of actin cytoskeleton	1.39	−1.49	2.36	*Blockade of Neurotransmitter Relase by Botulinum Toxin	0	1.48	3.68	*Glycine, serine and threonine metabolism*	−0.91	1.93	2.35
Fatty acid biosynthesis	1.33	2.3	1.1	*Glycosylphosphatidylinositol(GPI)-anchor biosynthesis*	0	0.96	−2.44	Hedgehog signaling pathway	−1.12	−1.65	2.24
Metabolism of xenobiotics by cytochrome P450	1.32	−2.37	2.78	*Atrazine degradation	0	0.48	2.78	*Endometrial cancer*	−1.28	2.27	2.52
*Neuroactive ligand-receptor interaction*	1.3	2.37	3.07	Retinol metabolism	0	0	2.55	Jak-STAT signaling pathway	−1.35	3.5	1.41
Phenylalanine metabolism	1.25	0	2.35	*Nitrogen metabolism	0	0	2.64	*Toll-like receptor signaling pathway*	−1.42	2.72	−1.83
*Tyrosine metabolism*	1.25	−1.65	2.76	Alanine and aspartate metabolism	0	−0.53	2.29	Alkaloid biosynthesis I	−1.42	0	2.35
*Calcium signaling pathway*	1.13	1.19	3	*Butanoate metabolism*	0	−0.69	2.97	Regulation of autophagy	−1.5	−1.11	−2.31
TGF-beta signaling pathway	1.12	−2.16	−3.13	Ether lipid metabolism	0	−0.88	2.36	*Citrate cycle (TCA cycle)*	−1.97	−2.95	0.93
Glycan structures - biosynthesis 1	1.02	1.12	2.73	Synthesis and degradation of ketone bodies	0	−1.19	2.35	*Melanogenesis*	−2.03	−2.04	2.77
*Cytokine-cytokine receptor interact.*	0.98	2.75	1.92	*Starch and sucrose metabolism*	0	−1.72	2.4	*Long-term potentiation*	−2.31	1.35	−3.09
*Folate biosynthesis*	0.94	−1.69	−2.43	*1- and 2-Methylnaphthalene degradation	0	−2.07	3.53	*One carbon pool by folate*	−2.46	1.63	−2.78
								Anthrax Toxin Mechanism of Action pathway	−2.83	−0.84	2.49

**Table 4:** Late Phase Significantly Perturbed Pathways Bayesian *z*-Scores.

Activated Pathway Description	t = 720	Activated Pathway Description (con't)	t = 720	Suppressed Pathway Description	t = 720
Fructose and mannose metabolism	3.54	Type I diabetes mellitus	2.74	*ABC transporters	−2.25
*Cytokine-cytokine receptor interaction*	3.4	DNA polymerase	2.73	*B cell receptor signaling pathway	−2.25
*Complement and coagulation cascades*	3.37	*N-Glycan biosynthesis	2.73	*Non-small cell lung cancer	−2.25
Hedgehog signaling pathway	3.3	RNA polymerase	2.73	*Chronic myeloid leukemia	−2.26
*Biosynthesis of steroids	3.29	Gap junction	2.72	*Melanoma	−2.28
Riboflavin metabolism	3.26	*Type II diabetes mellitus	2.72	*Renal cell carcinoma	−2.28
*Circadian rhythm	3.24	Inositol metabolism	2.71	*Biotin metabolism	−2.29
*Starch and sucrose metabolism*	3.23	*mTOR signaling pathway	2.7	*Terpenoid biosynthesis	−2.29
*Alzheimer's disease	3.22	*PPAR signaling pathway*	2.66	*VEGF signaling pathway	−2.31
*Melanogenesis*	3.21	ErbB signaling pathway	2.64	*Valine, leucine and isoleucine biosynthesis	−2.32
*One carbon pool by folate*	3.21	*Taste transduction	2.64	*Linoleic acid metabolism	−2.37
Wnt signaling pathway	3.15	*Tyrosine metabolism*	2.64	*Aminoacyl-tRNA biosynthesis	−2.39
TGF-beta signaling pathway	3.08	*Sphingolipid metabolism	2.63	*Glioma	−2.4
*Basal cell carcinoma*	3.07	*Cell adhesion molecules (CAMs)	2.56	Small cell lung cancer	−2.42
*Aminosugars metabolism	3.06	Phenylalanine metabolism	2.55	*Colorectal cancer	−2.43
*Cell Communication*	3.06	*Epithelial cell signaling Helicobacter pylori infection	2.53	Proteasome	−2.45
*Long-term potentiation*	3.06	*Phenylalanine, tyrosine & tryptophan biosynthesis	2.52	Propanoate metabolism	−2.5
*CD40L Signaling Pathway*	3.03	*Cholera	2.51	Lysine degradation	−2.51
*Hematopoietic cell lineage*	3.03	*Glutamate metabolism	2.51	Porphyrin and chlorophyll metabolism	−2.61
*Pyruvate metabolism*	3.03	Alkaloid biosynthesis I	2.49	Carbon fixation	−2.62
ECM-receptor interaction	3.02	*Pathogenic Escherichia coli infection*	2.49	*Citrate cycle (TCA cycle)*	−2.63
Olfactory transduction	3.01	*Acute myeloid leukemia*	2.48	Axon guidance	−2.64
*Neuroactive ligand-receptor interaction*	2.99	Stilbene, coumarine and lignin biosynthesis	2.48	*gamma-Hexachlorocyclohexane degradation	−2.64
Glycan structures - biosynthesis 1	2.98	Reductive carboxylate cycle (CO2 fixation)	2.47	*Leukocyte transendothelial migration	−2.7
Chondroitin sulfate biosynthesis	2.96	Alanine and aspartate metabolism	2.44	*D-Glutamine and D-glutamate metabolism	−2.73
*Folate biosynthesis*	2.96	GnRH signaling pathway	2.42	Cytokine Inflammatory Response	−2.77
*Pentose and glucuronate interconversions*	2.93	*Pyrimidine metabolism	2.41	*Cell cycle	−2.81
*Galactose metabolism	2.92	Parkinson's disease	2.39	*Tetrachloroethene degradation*	−2.82
Jak-STAT signaling pathway	2.91	Methionine metabolism	2.37	Oxidative phosphorylation	−2.88
Arachidonic acid metabolism	2.9	*Bile acid biosynthesis*	2.36	*microtubule-associated protein 1A*	−3.3
*Protein export	2.89	Peptidoglycan biosynthesis	2.36	*Thiamine metabolism	−3.3
*Endometrial cancer*	2.86	*Valine, leucine and isoleucine degradation	2.36		
Regulation of actin cytoskeleton	2.86	*Glycosylphosphatidylinositol(GPI)-anchor biosynthesis*	2.35		
*Toll-like receptor signaling pathway*	2.85	*MAPK signaling pathway	2.35		
*Butanoate metabolism*	2.84	*Prostate cancer	2.35		
C5-Branched dibasic acid metabolism	2.84	*Bladder cancer	2.33		
*Glycine, serine and threonine metabolism*	2.84	Inositol phosphate metabolism	2.33		
*Adipocytokine signaling pathway*	2.83	*Novobiocin biosynthesis	2.33		
*Glycolysis/Gluconeogenesis*	2.82	*Monoterpenoid biosynthesis	2.32		
*Glycerolipid metabolism	2.8	*Apoptosis	2.3		
*Calcium signaling pathway*	2.78	Thyroid cancer	2.29		
Purine metabolism	2.77	Regulation of autophagy	2.28		
Synthesis and degradation of ketone bodies	2.76	Arginine and proline metabolism	2.26		

### System Level Pathway Results and Immune Response Phases

**Figure 1:**
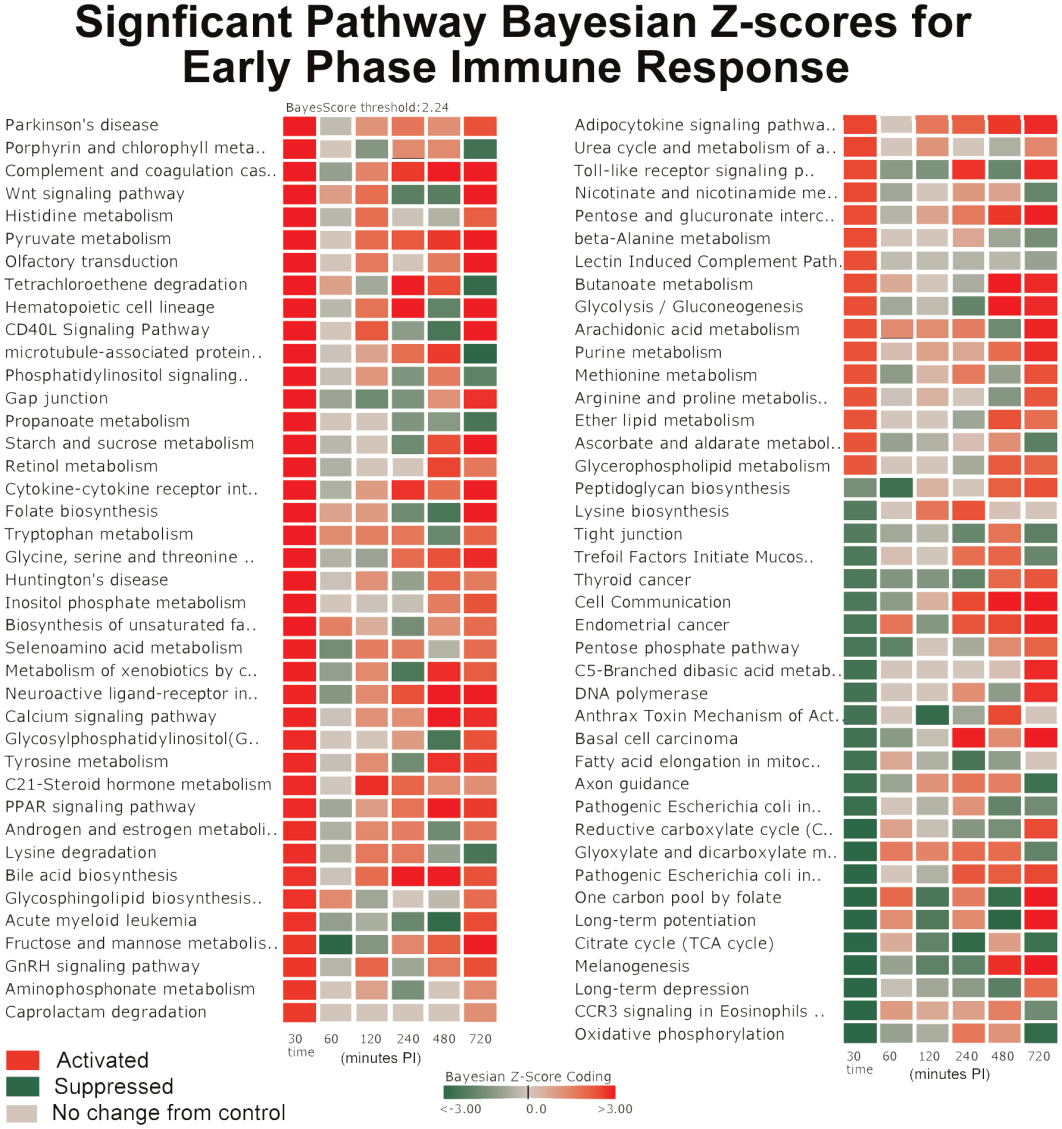
Significantly Perturbed Pathways of the Early Phase Immune Response. The darker red gradients indicate higher activation scores (more up-regulated gene expression within the pathway) while the darker green gradients indicate more suppressed pathway activity (more down-regulated gene expression) of MAP infected Peyer's patch. The pathway threshold score was selected for a 97.5% confidence.

There were 30 common pathways significantly perturbed in all three phases. These common pathways may be important to both short term and long term host tolerance to MAP. In the “common" pathways, there were several pathways involved in the host immune response including: Complement and Coagulation Cascades indicating a non-specific defense mechanism; Hematopoietic Cell Lineage indicating immune cell differentiation; CD40L Signaling indicating T cell activation; Cytokine-Cytokine Receptor indicating immune cell communication, PPAR Signaling indicating inflammatory response of immune cells; and Toll-Like Receptor Signaling that signifies triggering the innate immune response. These immune related pathways all had basically strong activation as shown in Table 2 for the Early Phase in Figure 1.

A few pathways that were apparently being manipulated by MAP infection showed a reversal from suppression in the Early Phase to activation in the Late Phase that include Cell Communication, One Carbon Pool by Folate, and Long-term Potentiation while the Microtubule-associated Protein 1 pathway reversed from activated to a suppressed state (Figure 1). Other pathways that were activated in all three phases include Complement and Coagulation Cascade pathway, Adipocytokine Signaling Pathway, Hematopoietic Cell Lineage Pathway, and Neuroactive ligand-receptor interaction. The genes and networks involved in these pathways are discussed in more detail in the Discussion section.

There are 23 pathways that are uniquely perturbed during the Early Phase of MAP infection and 26 non-unique pathways that are in common with either the Intermediate or Late Phases that include a number of immune and metabolic pathways as annotated in Tables 2, 3 and 4. These included for example, Tyrosine Metabolism, Histidine Metabolism, Phosphatidylinositol Signaling System, Tryptophan Metabolism, Selenoamino Acid Metabolism, Androgen and Estrogen Metabolism, Glycosphingolipid Biosynthesis, Aminophosphonate Metabolism, Glycerophospholipid Metabolism, GnRH Signaling Pathway, Ether Lipid Metabolishm, and Glycolysis/Gluconeogenesis.

The temporal perturbation of these pathways illustrates the complexity of MAP's pathogenicity in the host. The functional roles of these pathways with regard to host invasion and evasion are presented in more detail In the Discussion section. The analysis resulted in the development of a biological systems level model.

### Gene Ontology (GO) Biological Process Results

**Figure 2:**
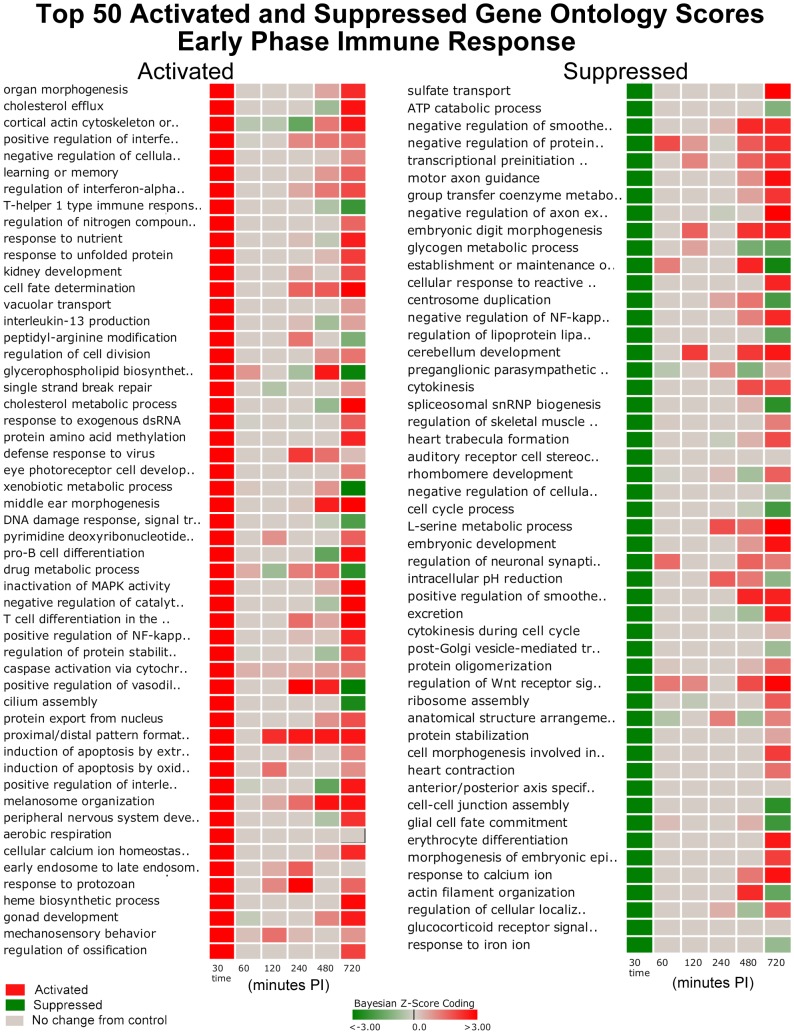
Top 50 Activated and Suppressed Perturbed Gene Ontology Scores Early Phase Response. The darker red gradients indicate higher activation scores (more up-regulated gene expression within the pathway) while the darker green gradients indicate more suppressed GO activity (more down-regulated gene expression) of MAP infected Peyer's patch. The pathway threshold score was selected for a 97.5% confidence.

The DBGGA scoring method was applied to 2,254 GO biological process categories. Each category had to contain at least 10 measured genes to be scored. For GO scoring, the DBGGA method employs a naive Bayesian classifier network model. Scoring results indicated that the Early Phase response had 467 significantly perturbed GO categories (Table S1), the Intermediate Phase had 105 (Table S2), and the Late Phase had 691 (Table S3). There were 27 GO categories that were significantly perturbed in common to all three phases. In the Early Phase there were 272 uniquely perturbed GO categories while the Intermediate Phase had 31 and the Late Phase had 459 that were uniquely perturbed. There is a broad range of strongly activated and suppressed biological processes. Several GO categories of interest were selected from the Early Phase for heat map plotting shown in Figure 2. Of the top 50 GO activated categories, early Phase activation may be associated with MAP host invasion and early immune defense processes. In contrast, of the top 50 suppressed GO categories, the suppressed categories may be a result of MAP's manipulation of these host's processes to facilitate invasion into the host cells and to subvert the host's immune defenses.

Several activated GO categories of interest include processes involved in: 1) early to late endosome transport; 2) cellular calcium ion homeostasis; 3) induction of apoptosis; 4) positive regulation of NF-κB transcription factor activity; 5) inactivation of MAPK activity; 6) vacuolar transport; 7) Wnt receptor signaling; 8) actin filament bundle formation; 9) defense response to Gram-positive bacterium; and 10) activation of phospholipase C activity. These categories all had an activated state in the Early Phase of the immune response (Figure 2). Interestingly, there were six GO categories that reversed from activated state in the Early Phase to a suppressed state in the Late Phase that included: 1) positive regulation of vasodilation; 2) T-helper 1 type immune response; 3) complement activation, alternative pathway; 4) regulation of Rab GTPase activity; 5) response to toxin; and 6) activation of transmembrane receptor protein tyrosine kinase activity.

Several interesting GO categories that were suppressed in the Early Phase and then reversed to an active state in the Late Phase include processes involved in: 1) I-κB kinase/NF-κB cascade; 2) actin cytoskeleton reorganization; 3) Wnt receptor signaling pathway through beta-catenin; 4) T cell differentiation; 5) microtubule-based movement; 6) positive regulation of endocytosis; 7) positive regulation of apoptosis; 8) cytoskeletal anchoring at plasma membrane; 9) response to calcium ion; 10) negative regulation of NF-κB transcription factor activity; and 11) negative regulation of axon extension. Other categories that were suppressed in the Early Phase and remained suppressed or neutral included: 1) Rho protein signal transduction; 2) positive regulation of Wnt receptor signaling pathway; 3) T cell proliferation; 4) regulation of actin filament polymerization; 5) cell-cell junction assembly; 6) post-Golgi vesicle-mediated transport; and 7) regulation of lipoprotein lipase activity.

There were 554 GO categories that were strongly activated in the Late Phase (Table S3). These activated categories provided evidence of the host mounting a more effective immune response that included: 1) neutrophil activation; 2) positive regulation of inflammatory response; 3) innate immune response; 4) positive regulation of B cell proliferation; 5) positive regulation of T cell mediated immunity; 6) endothelial cell proliferation; 7) positive regulation of cytokine production; and 8) positive regulation of cell adhesion. In contrast, there were only 130 GO categories that were significantly suppressed in the Late Phase that included: 1) immunoglobulin mediated immune response; and 2) natural killer cell activation.

### Discovery of Mechanistic Genes during MAP Infection Reveals Importance of the Cross-Talk via Inter-Pathway Interactions

The DBGGA analysis identifies mechanistic genes by Bayesian modeling of the genes in the context of their upstream and downstream relationships over the complete time course. Mechanistic genes significantly influenced disease progression and contributed most to the discrepancies between the MAP infected vs. PBS control tissue. We propose that these mechanistic genes may play an important role in the outcome of the host-pathogen interaction. A complete list of all the mechanistic genes is provided in the Table S4. We further focused on only 43 pathways involved in signaling and immune response. Our intent was to identify mechanistic genes that are associated in multiple pathways which may be the source of cross-talk and thus have more significant influence governing the host immune tolerance to MAP and illustrating the importance of cross-talk. Of the 43 pathways analyzed, 36 pathways had at least one overlapping mechanistic gene. It was found that 141 mechanistic genes had overlaps within the 36 pathways examined. These genes are listed in Table S5. Of highest interest were those genes that had influence (overlap) across numerous pathways. The mechanistic gene, AKT2 (v-akt murine thymoma viral oncogene homolog 2), had overlap with 11 important pathways that include: 1) Adipocytokine signaling; 2) Insulin signaling; 3) Fc epsilon RI signaling; 4) T cell receptor signaling; 5) Toll-like receptor signaling; 6) Tight junction; 7) Integrin-mediated cell adhesion; 8) VEGF signaling; 9) mTOR signaling; 10) ErbB signaling; and 11) MAPK signaling. These pathways are involved in a wide variety of biological processes including, but not limited to cell proliferation, differentiation, apoptosis, tumorogenesis, as well as glycogen synthesis and glucose uptake. The protein encoded by *AKT2* is a member of the AKT, also called PKB, serine/threonine protein kinase family. AKT kinases play a key role in regulating cell survival, insulin signaling, angiogenesis and tumor formation. Only 14 other mechanistic genes had overlap with 6 or more pathways as listed in Table S5. Several cytokines (*IL1a*, *IL1ß*, *IL4R*, *IL5*, *IL7*, *IL15*, *IL23A*, *IFN-γ*) were also overlapping mechanistic genes in analyzed pathways. These genes will be addressed in more detail in the Discussion section.

## Discussion

MAP has the uncanny ability to persist within the host for an indefinite period of time that can last several years. Hence, MAP must have efficient host invasion and host immune evasion processes that should be evident by MAP's manipulation of certain host immune response and metabolic pathways. We utilized the perinatal calf ligated jejunal-ileal loop model to study the sequential changes in the host intestine immediately after infection with MAP. A key role of intestinal mucosal epithelia is barrier function, which prevents colonization or invasion by foreign microorganisms. However, in Johne's disease, MAP invade M cells, enterocytes, dendritic cells and macrophages, and are capable of resisting host defenses and multiply to reach very high intracellular numbers leading to chronic granulomatous lesions [31], [32]. In infected subclinical and clinically affected animals, systemic immune response is achieved. Persistence of the organism in the intestinal Peyer's patch in the presence of a systemic immune response suggests that the immune response in the intestine may be fundamentally different from the systemic response. In fact, a state of immune tolerance was detected at the mucosal level during subclinical Johne's disease [7]. Furthermore, it has been shown earlier that the human intestinal macrophages display profound inflammatory anergy despite avid phagocytic and bacteriological activity [33]. The aim of our study was to discover if the immune tolerance is initiated, and if so, how quickly after the pathogen comes in contact with the intestinal mucosa. We hypothesized from a biological system perspective that MAP pathogenicity should show evidence of: 1) host invasion by manipulating host cellular functions related to mucosal immune barrier; and 2) subversion of host immune response that permits MAP uptake, survival and proliferation.

### Host Invasion through Compromising the Mucosal Immune Barrier

**Figure 3:**
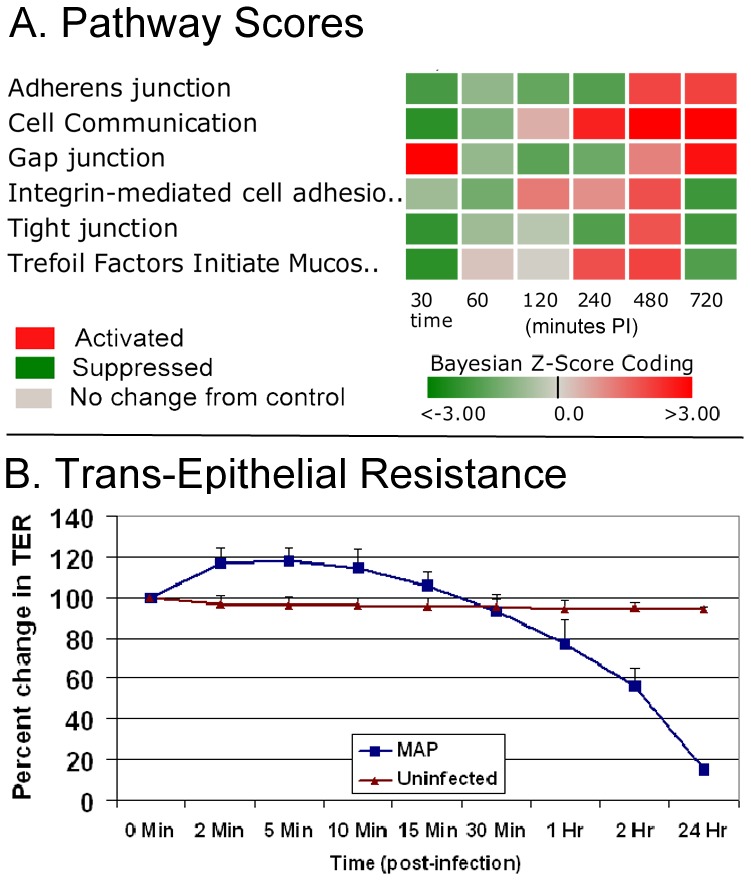
Pathway Scores for Cell Communication and Cell Adhesion Pathways and their Involvement in Trans-Epithelial Resistance (TER). The set of genes within the gap junction, tight junction, adherens junction and integrin-mediated cell adhesion are contained in the cell communication pathway. (3a). Pathway scores for Adherens Junction (AJ), Cell Communication (CC), Gap Junction (GJ), Integrin-mediated Cell Adhesion (IMCA), Tight Junction (TJ) and Trefoil Factors Initiated Mucosal Healing (TFIMH). Red indicates an activated state while green indicates suppression. Note the reversal of the cell communication pathway from suppressed to activated from the Phase 1 (30–60 min.) to the Phase 3 time period (720 min.). (3b). Changes in the Trans-Epithelial Resistance of polarized T-84 cells exposed to MAP for 24 h. Data represent mean ± SD from 3 measurements at each time point and three independent experiments.

In the pathway scores listed in Tables 2, 3 and 4, there are several suppressed pathways that may be associated with MAP host invasion by impeding mucosal epithelial barrier function that include Cell Communication (CC), Tight Junction (TJ), Integrin-mediated Cell Adhesion (IMCA), and Trefoil Factors Initiated Mucosal Healing (TFIMH) pathways. A key observation is the suppressed state of the Cell Communication Pathway, which interestingly was suppressed in the Early Phase and became activated in the Late Phase (Figure 1). The CC pathway includes the genes from the TJ, IMCA as well as the Gap Junction (GJ) and Adherens Junction (AJ) pathways. These pathways form the intercellular junction complexes between adjacent intestinal epithelial cells that are critical components of the intestinal mucosal barrier that creates a semi-permeable diffusion barrier. Studies have shown that activation (increased gene expressions) of these junction pathways may lead to strengthening the intestinal barrier while suppression (disrupted gene expression) may result in weakening of the immune barrier. As shown in the heat map scores of Figure 3(a), the AJ, TJ, and TFIMH pathways are suppressed in the Early Phase while the state of the Gap Junction pathway was activated. This suggests that MAP host invasion may be disrupting critical cell communication processes in a complex manner. This complex nature of cell disruption was also analyzed by measuring the Trans-Epithelial Resistance (TER) of an *in vitro* model polarized epithelial cells during MAP interaction (Figure 3(b)). MAP infection caused a marked decrease in the TER, adding credibility that increased permeability of *in vivo* host intestinal epithelium may facilitate bacterial invasion through the intestinal epithelium.

Cell adhesion serves to facilitate trafficking and migration of T lymphocytes into sites of inflammation, movement of lymphocytes within the rich environment found in extravascular tissue, and the physical interaction between antigen-reactive T cells and antigen-presenting cells that is required for efficient T-cell activation [34]. As shown in Figure 3(a), the IMCA and TFIMH pathways are suppressed in the early and Late Phases which suggest that MAP may disrupt T lymphocyte recruitment that helps explain the lack of chronic inflammation observed in the MAP infected ileal loops and subvert mucosal healing. Over time the trend is for the TJ, IMCA and TFIMH pathways to remain suppressed, but GJ and AJ pathways become activated. This suggests that MAP may need to suppress important host cell communication, adhesion and healing processes for penetrating the mucosal immune barrier, but activate cell adhesion mechanisms for longer term survival in the Late Phase. It has been proposed by others [35] that some bacteria survival mechanism in mucosal epithelial cells is for the bacteria to hijack integrin-linked kinase to stabilize focal adhesions and block cell detachment of infected cells. The rapid turnover and exfoliation of mucosal epithelial cells provides an innate defense system against bacterial infection. Nevertheless, bacteria such as MAP may be able to subvert this immune defense mechanism and colonize the epithelium efficiently and survive. Furthermore, M cells are unique among cells of the intestinal epithelium as they display a high density of Beta1 integrins on their luminal surface. In a recent study, we documented that the early host response was evident by the presence of MAP in the vicinity of M cells and goblet cells [2]. Integrins have affinity for the fibronectin attachment protein of MAP. Thus, M cells are thought to play a role in the host defense by down-regulating integrins and thus avoiding the fibronectin bridge formation for the entry of MAP into the ileal mucosa [36].

#### Junction (Gap, Tight, Adherens) Pathways

**Figure 4:**
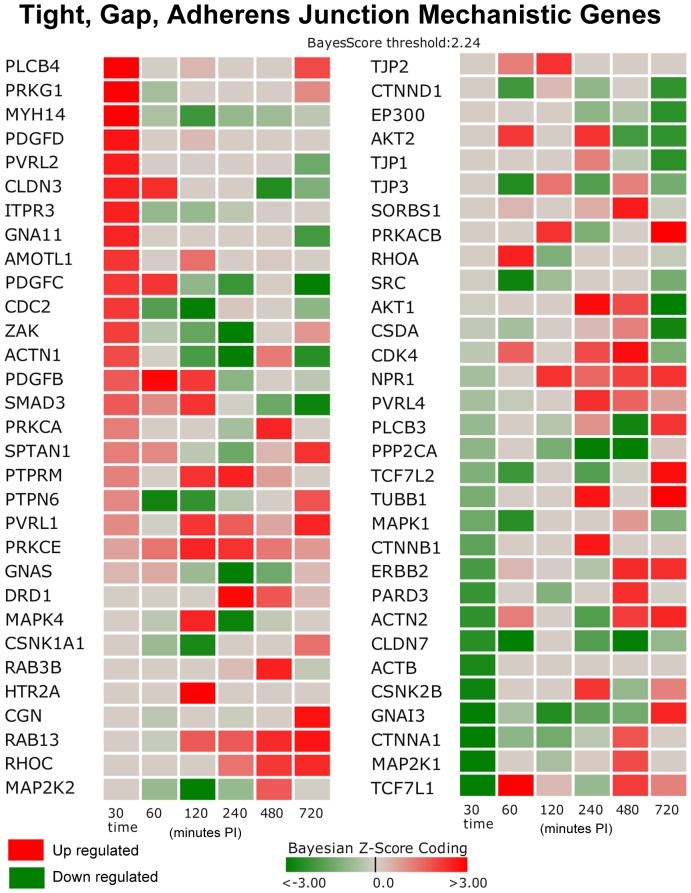
Heat Map of Mechanistic Genes for Junction (Adherens, Gap, and Tight) Related Pathways. The junction related mechanistic genes determined significant by DBGGA analysis. The heat map shows a dominance of down regulated junction related gene expression occurring in the Early Phase as indicated by the darker green boxes. Genes listed surpassed the |Bayesian z-score|>2.24 at any of the time points. Red indicates up regulation while green indicates down regulation. Time is minutes post-infection.

The junction related mechanistic genes (significant differential gene expressions determined by DBGGA analysis) are shown in the heat map of Figure 4. The key down-regulated genes of high interest in the Early Phase include: *MAPK1*, *CTNNB1*, *ERBB2*, *PARD3 ACTN2*, *CLDN7*, *ACTB*, *CSNK2B*, *CSNK2B*, *GNAI3*, *MAP2K1*, *TCF7L1*, *SRC* and whose biological roles are described in Table 5. Many of these genes are involved with maintaining the integrity of the epithelial layer. According to the Adherens Junction Bayesian network model (not shown), *SRC* has strong correlated relationships with other downstream genes, i.e., the gene relationship SRC->RAC1. *RAC1* (ras-related C3 botulinum toxin substrate 1 rho family small GTP binding protein Rac1) gene expression is suppressed across all three phases. *RAC1* encodes a GTPase protein belonging to the RAS superfamily of small GTP-binding proteins that regulate a diverse array of cellular events including the control of cell growth, cytoskeletal reorganization, and the activation of protein kinases.

**Table 5:** Junction (Gap, Tight, Adherens) Pathways Mechanistic Genes.

*MAPK1*	mitogen-activated protein kinase 1	a gene encoding a member of the MAP kinase family known as extracellular signal-regulated kinases (ERKs) which act as an integration point for multiple biochemical signals, and are involved in a wide variety of cellular processes such as proliferation, differentiation, transcription regulation, and development
2) *CTNNB1*	catenin beta 1	a gene encoding a protein necessary for the creation and maintenance of epithelial cell layers by regulating cell growth and adhesion between cells and also anchors the actin cytoskeleton
*ERBB2*	v-erb-b2 erythroblastic leukemia viral oncogene homolog 2	encodes a member of the epidermal growth factor (EGF) receptor family of receptor tyrosine kinases;
*PARD3*	par-3 partitioning defective 3 homolog	gene encodes an adapter protein involved in asymmetrical cell division and cell polarization processes and plays a central role in the formation of epithelial tight junctions
*ACTN2*	actinin, alpha 2	encodes a cytoskeletal protein that anchors actin to a variety of intracellular structures
*CLDN7*	claudin 7	encodes a member of the claudin family which includes integral membrane proteins and components of tight junction strands that serve as a physical barrier to prevent solutes and water from passing freely through the paracellular space between epithelial or endothelial cell sheets and also play critical roles in maintaining cell polarity and signal transductions
*ACTB*	actin, beta	encodes an actin protein involved in cell motility Structure and integrity and is a major constituent of the contractile apparatus
*CSNK2B*	casein kinase 2	gene encodes a beta subunit of casein kinanse II, a serine/threonine protein kinase that is thought to have a regulatory function in cell proliferation, cell differentiation, and apoptosis and also thought to influence Wnt Signaling via beta-catenin phosphorylation and the PI 3-K signaling pathway via the phosphorylation of Akt
*GNAI3*	guanine nucleotide binding protein (G protein)	gene encodes a protein that is a membrane bound GTPase that is linked to 7-TM receptors
*MAP2K1*	mitogen-activated protein kinase kinase 1	gene encodes a protein that is a member of the dual specificity protein kinase family which acts as a mitogen-activated protein (MAP) kinase kinase and is an integration point for multiple biochemical signals
*TCF7L1*	transcription factor 7-like (T-cell specific HMG-box)	a transcription factor activated by beta catenin and known to mediate the Wnt Signaling pathway
*SRC*	v-src sarcoma viral oncogene	encodes the tyrosine-protein kinase protein that plays a role in cell growth. According to the Adherens Junction Bayesian network model the gene relationship *SRC*->*RAC1*. *RAC1* gene expression was suppressed across all three phases. *RAC1* (ras-related C3 botulinum toxin substrate 1 rho family small GTP binding protein Rac1) encodes a GTPase protein belonging to the RAS superfamily of small GTP-binding proteins that regulate a diverse array of cellular events including the control of cell growth cytoskeletal reorganization and the activation of protein kinases

#### Cell Adhesion Molecules (CAM) and Integrin-Mediated Cell Adhesion (IMCA) Pathway

**Figure 5:**
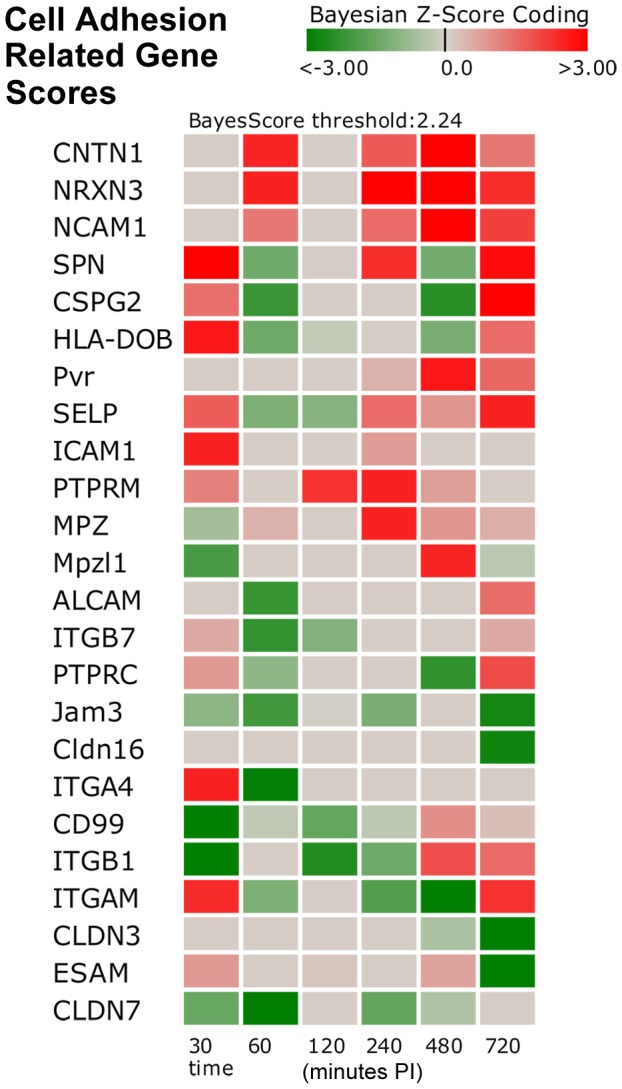
Cell Adhesion Related Gene Scores. The heat map shows a dominance of higher perturbed cell adhesion gene expression (both up- and down-regulated) occurring in the Early Phase as indicated by the darker red and green boxes for time t = 30 and t = 60 minutes. Genes listed surpassed the |Bayesian z-score|>2.24 at any of the time points. Red indicates an activated state while green indicates suppression. Time is minutes post-infection.

The impairment of cell adhesion may be an important mechanism for MAP invasion in the Early Phase as evident by the IMCA pathway suppression, while the strong Late Phase activation of CAM pathway may be a MAP survival mechanism which prevents infected cell detachment. To explore this in more detail, the gene level activities in the Early, Intermediate, and Late Phases were examined (Figure 5). An important gene, *CDH5* (cadherin 1, type 1, E-cadherin (epithelial)), of epithelial cells that form an adhesion point for many subtypes of lymphocytes including intraepithelial lymphocytes lacked expression or was slightly down-regulated in all phases. *Integrins* function in neutrophil adherence but the majority of integrins was down regulated or not expressed in the Early Phase. The Intermediate and Late Phases had a greater number of up regulated integrins that may support the strengthening of the immune barrier. In the CAM and IMCA pathway there were nine strongly down-regulated genes in the Early Phase that supports impaired cell adhesion, i.e. mucosal barrier weakening. These genes include *ITGB1*, *PTK2*, *MAP2K1*, *SELL*, *MAPK1*, *Mpzl1*, *CD99*, *ITGA4* and *CLDN7* and are described in Table 6. Note that *CLDN7* was described above as an integral membrane protein and component of tight junction as was the role of *MAPK1*. The key Intermediate and Late Phase up-regulated genes in the CAM and IMCA pathways, in support of MAP survival mechanism (mucosal barrier strengthening) are *PDPK1*, *CNTN1*, *NRXN3*, *SPN*, *CSPG2*, *HLA-DOB*, *SELP*, *PTPRC*, *ITGAM*, *TLN1*, *NCAM1*, and *RHOC*. Detailed description of these genes is provided in Table 7.

**Table 6:** Key Down-Regulated Mechanistic Genes for Cell Adhesion and Integrin-Mediated Cell Adhesion Pathways.

*ITGB1*	integrin, beta 1 (fibronectin receptor)	encodes an integrin membrane receptor involved in cell adhesion and a number of processes including immune response
*PTK2*	protein tyrosine kinase 2	encodes cytoplasmic protein tyrosine kinase which is found concentrated in the focal adhesions
*MAP2K1*	mitogen-activated protein kinase kinase 1	encodes a member of the dual specificity protein kinase family that lies upstream of MAP kinases and stimulates the enzymatic activity of MAP kinases upon wide variety of extra- and intracellular signals
*SELL*	selectin L (lymphocyte adhesion molecule 1	encodes a cell surface adhesion molecule that belongs to a family of adhesion/homing receptors
*MAPK1*	mitogen-activated protein kinase 1	a gene encoding a member of the MAP kinase family known as extracellular signal-regulated kinases (ERKs) which act as an integration point for multiple biochemical signals, and are involved in a wide variety of cellular processes such as proliferation, differentiation, transcription regulation, and development
*Mpzl1*	myelin protein zero-like 1	encodes a cell surface receptor and is believed to be involved in regulation of integrin-mediated cell motility
*CD99*	CD99 molecule	encodes a cell surface glycoprotein involved in leukocyte migration, T-cell adhesion, ganglioside GM1 and transmembrane protein transport, and T-cell death by a caspase-independent pathway
*ITGA4*	integrin, alpha 4	encodes an integrin alpha chain family protein integral to the membrane and known to be involved in cellular adhesion/aggregation processes
*CLDN7*	claudin 7	encodes a member of the claudin family which includes integral membrane proteins and components of tight junction strands that serve as a physical barrier to prevent solutes and water from passing freely through the paracellular space between epithelial or endothelial cell sheets and also play critical roles in maintaining cell polarity and signal transductions

**Table 7:** Up-Regulated Mechanistic Genes for Cell Adhesion Pathway in Intermediate and Late Phases.

*PDPK1*	3-phosphoinositide dependent protein kinase-1	May play a role in signaling processes and phosphorylates and activates PKB/AKT.
*CNTN1*	contactin 1	Encodes a member of the immunoglobulin superfamily proteins and is an anchored neuronal membrane protein which functions as a cell adhesion molecule
*NRXN3*	neurexin 3	Encodes a protein that functions in the vertebrate nervous system as cell adhesion molecules
*SPN*	sialophorin *CSPG2*	Encodes major glycoproteins of thymocytes and T lymphocytes and plays a role in the physicochemical properties of the T-cell surface and in lectin binding, and in some B lymphocytes, where it appears to be important for immune function and may be part of a physiologic ligand-receptor complex involved in T-cell activation
*CSPG2*	Chondroitin sulfate proteoglycan 2	Encodes a member of the aggrecan/versican proteoglycan family protein involved in cell adhesion, proliferation, migration and angiogenesis and may play a role in intercellular signaling and in connecting cells with the extracellular matrix
*HLA-DOB*	major histocompatibility complex class II, DO beta	encodes a MHC class II protein that is an important modulator in the antigen presentation pathway by interaction with the HLA-DM molecule in B cells
*SELP*	selectin P, antigen CD62	Encodes a protein important for leukocyte-endothelial cell adhesion and mediates the interaction of activated endothelial cells or platelets with leukocytes
*PTPRC*	protein tyrosine phosphatase, receptor type, C	Encodes a member of the protein tyrosine phosphatase (PTP) family. PTPs are known to be signaling molecules that regulate a variety of cellular processes, including cell growth, differentiation, mitotic cycle, and oncogenic transformation and is an essential regulator of T- and B-cell antigen receptor signaling
*ITGAM*	integrin, alpha M (complement component 3 receptor 3 subunit)	Encodes the protein integrin alpha-M/beta-2 and is implicated in various adhesive interactions of monocytes, macrophages and granulocytes as well as in mediating the uptake of complement-coated particles
*TLN1*	talin 1	encodes a cytoskeletal protein that is concentrated in areas of cell-substratum and cell-cell contacts and plays a significant role in the assembly of actin filaments and in spreading and migration of various cell types, including fibroblasts and osteoclasts
*NCAM1*	neural cell adhesion molecule 1	Encodes a cell adhesion protein that is a member of the immunoglobulin superfamily that is involved in cell-to-cell interactions as well as cell-matrix interactions during development and differentiation. This protein has been shown to also be involved in development of the nervous system, and for cells involved in the expansion of T cells and dendritic cells which play an important role in immune surveillance receptors
*RHOC*	ras homolog gene family, member C	encodes a member of the Rho family of small GTPases, which cycle between inactive GDP-bound and active GTP-bound states and function as molecular switches in signal transduction cascades. Rho proteins promote reorganization of the actin cytoskeleton and regulate cell shape, attachment, and motility

#### Trefoil Factors Initiated Mucosal Healing (TFIMH) Pathway

Epithelial continuity can also depend on a family of small, yet abundant, secreted proteins–the trefoil factors. The immune related TFIMH pathway is suppressed in the Early Phase (Figure 3a). The trefoil factors maintain the integrity of the gastrointestinal tract, despite the continual presence of microbial flora and injurious agents [37]. Unfortunately, the trefoil factors gene probes were not included on the bovine microarray employed during this study. However, the TFIMH pathway suppression (as determined by other observed gene expressions) could imply impaired trefoil factors gene expression, and consequently, a possible invasion mechanism of MAP by subverting mucosal healing. Genes that dominate the suppressed pathway activity are *PTK2*, *ITGB1*, *MAPK1* and *CTNNB1*. The biological roles of these genes are described in Table 8.

**Table 8:** Key Down-Regulated Mechanistic Genes of the Trefoil Factors Initiated Mucosal Healing Pathway.

*PTK2*	protein tyrosine kinase 2	Encodes a cytoplasmic protein tyrosine kinase that is found concentrated in the focal adhesions that form between cells growing in the presence of extracellular matrix constituents. The encoded protein is a member of the FAK subfamily of protein tyrosine kinases.
*ITGB1*	integrin, beta 1 (fibronectin receptor, beta polypeptide, antigen CD29)	Encodes the integrin beta 1 protein which is a membrane receptor involved in cell adhesion and recognition in a variety of processes including embryogenesis, hemostasis, tissue repair, immune response and metastatic diffusion of tumor cells.
*MAPK1*	mitogen-activated protein kinase 1	Agene encoding a member of the MAP kinase family known as extracellular signal-regulated kinases (ERKs) which act as an integration point for multiple biochemical signals, and are involved in a wide variety of cellular processes such as proliferation, differentiation, transcription regulation, and development
*CTNNB1*	catenin beta 1	Agene encoding a protein necessary for the creation and maintenance of epithelial cell layers by regulating cell growth and adhesion between cells and also anchors the actin cytoskeleton

### Subversion of Host Immune Response Processes

#### Host Cellular Uptake of MAP and Phagocytosis Arrest

A new perspective in the pathogenesis on mycobacterial diseases (*M. tuberculosis*) is the exploitation of host cell signaling pathways by the pathogen. Upon infection, the phosphatases and kinases of several pathogenic bacteria modify host proteins and help in the establishment of the disease. The uptake of *M. tuberculosis* by macrophages is associated with a number of Early Phase signaling events, such as the recruitment and activation of members of the Src family of protein tyrosine kinases [38]. These kinases result in the increased tyrosine phosphorylation of multiple macrophage proteins and the activation of phospholipase D [39]. Phospholipase products have been linked to phagocytosis mechanisms of bacteria uptake [40]. Examination of the pathways that include *CSK* (c-src tyrosine kinase) indicated that this gene is significantly up-regulated in the Early Phase and transitioned to a moderately down-regulated state in the Intermediate and Late Phases. *CSK* is associated with the Regulation of Actin Cytoskeleton, Epithelial Cell Signaling, Integrin-mediated Cell Adhesion, and Activation of Csk Through T-Cell Receptor pathways, all of these pathways were highly activated in the Early Phase and transitioned to suppressed states in the Intermediate and Late Phases. In this study, several classes of phospholipases were significantly up-regulated in the Early Phase that included *PLA2G1B* (phospholipase A2, group IB (pancreas)), *PLCD1* (phosholipase C, delta 1), *PLCB4* (phospholipase C, beta 4), and *PLD1* (phospholipase D1, phosphatidylcholine-specific). Table 9 lists the pathways in which these genes are considered mechanistic. Phospholipases are a group of enzymes that hydrolyze phospholipids into fatty acids and other lipophilic molecules and have been implicated in numerous cellular pathways, including signal transduction, membrane trafficking, and the regulation of mitosis. Elevated levels of phospholipases have been linked to intracellular calcium elevations during bacteria invasion [41].

**Table 9:** Up-Regulated Phospholipase Mechanistic Genes and Their Pathway Overlaps.

Gene	Pathway Overlaps
*PLA2G1B*	GnRH Signaling, Longterm Depression, VEGF Signaling, MAPK Signaling
*PLCB4*	GnRH Signaling, Longterm Depression, Phosphatidylinositol Signaling System, Calcium Signaling, Gap Junction, Wnt Signaling
*PLD1*	GnRH Signaling, Glycerophospholipid metabolism
*PLCD1*	Phosphatidylinositol Signaling System, Calcium Signaling

It has been shown that *M. tuberculosis* is able to hi-jack lipid metabolism to drive the progression of the disease [39], [42], [43]. The Phosphatidylinosital Signaling System (PSS) is of interest, because phosphatidylinositol lipids have been identified as key signaling mediators for random cell migration as well as chemoattractant-induced directional migration. The PSS was initially highly activated and trended to be suppressed in the Late Phase.

#### Phosphatidylinosital Signaling System (PSS) Pathway

The significantly up-regulated genes involved in this signaling event were *PLCD1*, *PLCB4*, *INPP4A*, *ITPR2*, *ITPR3*. The genes, PLCD1 and PLCB4 genes encode phospholipases that are ubiquitously expressed and have diverse biological functions including roles in inflammation, cell growth, signaling and death and maintenance of membrane phospholipids. Significantly down-regulated in all immune response phases in PSS is the gene *CALM2* (calmodulin 2) that is known to mediate the control of a large number of enzymes and other proteins by Ca^++^. The biological roles of these genes are described in Table 10.

**Table 10:** Mechanistic Genes of the Phosphatidylinositol Signaling System.

**Up-Regulated Mechanistic Genes**		
*PLCD1*	phospholipase C, delta 1	Encodes phosphoinositide-specific phospholipase C that acts as a signal transducer and has diverse biological functions including roles in inflammation, cell growth, signaling and death and maintenance of membrane phospholipids
*PLCB4*	phospholipase C, beta 4	The protein encoded by this gene catalyzes the formation of inositol 1,4,5-trisphosphate and diacylglycerol from phosphatidylinositol 4,5-bisphosphate. This reaction uses calcium as a cofactor and plays an important role in the intracellular transduction of many extracellular signals
*INPP4A*	inositol polyphosphate-4-phosphatase, type I	Encodes an Mg^++^ independent enzyme that hydrolyzes the 4-position phosphate from the inositol ring of phosphatidylinositol 3,4-bisphosphate, inositol 1,3,4-trisphosphate, and inositol 3,4-bisphosphate. This reaction uses calcium as a cofactor and plays an important role in the intracellular transduction of many extracellular signals similar to *PLCD1*
*ITPR2*	inositol 1,4,5-trisphosphate receptor, type 2	Encode receptors for inositol 1,4,5-trisphosphate, a second messenger that mediates the release of intracellular calcium
*ITPR3*	inositol 1,4,5-trisphosphate receptor, type 3	Encode receptors for inositol 1,4,5-trisphosphate, a second messenger that mediates the release of intracellular calcium
**Down-Regulated Mechanistic Genes**		
*CALM2*	calmodulin 2	The protein encoded by *CALM2* mediates the control of a large number of enzymes and other proteins by Ca^++^ and is involved in a genetic pathway that regulates the centrosome cycle and progression through cytokinesis

It has been observed elsewhere [44] that pathogenic mycobacteria (human macrophages infected with *Mycobacterium avium* subsp. *hominissuis*) have been shown to interfere with Ca^++^ and PI3K signaling pathways which are essential pathways for phagosomal maturation that requires *CALM2* activation. The *CALM2* gene expression data, from the referenced human macrophage study, was consistently down-regulated at all measured time points along with markedly reduced *STX3* (syntaxin 3) expression.

#### Microtubule-Associated Protein 1 (M-AP1) Pathway

Syntaxins are included in the M-AP1 pathway that was highly activated in the Early Phase. The protein encoded by *STX3* is a member of the syntaxin family of cellular receptors for transport vesicles that participate in exocytosis in neutrophils. Other members of the syntaxin family have been associated with *M. tuberculosis* phagosome maturation arrest [45]. This pathway reversed from a highly activated state to a highly suppressed state in Late Phase (Figure 1). It has been observed in murine macrophages that mycobacteria arrest the maturation of the early endosome to a phagolysosome by inhibiting fusion of the mycobacterium-containing phagosome with lysosomes [46]–[48]. The M-AP1 pathway activation reversal may suggest an important mechanism for MAP host immune evasion. In the M-AP1 pathway genes, *SNAP23* and *Vamp2*, were highly up-regulated, while the genes, *Vti1a* and *YKT6*, were strongly down-regulated in the Early Phase. In the Late Phase, there were five strongly down-regulated genes that dominate the suppression of M-AP1 pathway. These down-regulated mechanistic genes include *Vti1b*, *STX8*, *STX10*, *YKT6*, *STX6* and *GOSR2*. The *STX* genes are members of the syntaxin family involved in protein trafficking from early to late endosomes via vesicle fusion and exocytosis. The biological roles of these genes are described in Table 11.

**Table 11:** Mechanistic Genes of the Microtubule-Associated Protein 1 Pathway.

**Early Phase Up-Regulated Mechanistic Genes**		
*SNAP23*	synaptosomal-associated protein, 23 kDa	Encodes a protein structurally and functionally similar to SNAP25 and binds tightly to multiple syntaxins and synaptobrevins/VAMPs. It is an essential component of the high affinity receptor for the general membrane fusion machinery and is an important regulator of transport vesicle docking and fusion.
*Vamp2*	vesicle-associated membrane protein 2	Encodes a protein that is a member of the vesicle-associated membrane protein (VAMP)/synaptobrevin family. Synaptobrevins/VAMPs, syntaxins, and the 25-kD synaptosomal-associated protein SNAP25 are the main components of a protein complex involved in the docking and/or fusion of synaptic vesicles with the presynaptic membrane. This gene is thought to participate in neurotransmitter release at a step between docking and fusion.
**Early Phase Down-Regulated Mechanistic Genes**		
*Vti1a*	vesicle transport through interaction with t-SNAREs homolog 1A	V-SNARE that mediates vesicle transport pathways through interactions with t-SNAREs on the target membrane. These interactions are proposed to mediate aspects of the specificity of vesicle trafficking and to promote fusion of the lipid bilayers. Involved in vesicular transport from the late endosomes to the trans-Golgi network
*YKT6*	YKT6 v-SNARE homolog (S. cerevisiae)	Encodes a protein that is a member of the SNARE recognition molecules implicated in vesicular transport between secretory compartments. Functions in endoplasmic reticulum to Golgi transport; as part of a SNARE complex composed of GOSR1, GOSR2 and STX5. Functions in early/recycling endosome to TGN transport; as part of a SNARE complex composed of BET1L, GOSR1 and STX5.
**Late Phase Down-Regulated Mechanistic Genes**		
*Vti1b*	vesicle transport through interaction with t-SNAREs homolog 1B	V-SNARE that mediates vesicle transport pathways through interactions with t-SNAREs on the target membrane. These interactions are proposed to mediate aspects of the specificity of vesicle trafficking and to promote fusion of the lipid bilayers. May be concerned with increased secretion of cytokines associated with cellular senescence.
*STX8*	CIP-1-associated regulator of cyclin B	The gene is a member of the syntaxin family. The encoded protein is involved in protein trafficking from early to late endosomes via vesicle fusion and exocytosis.
*STX10*	syntaxin 10	SNARE involved in vesicular transport from the late endosomes to the trans-Golgi network
*STX6*	syntaxin 6	Involved in intracellular vesicle trafficking
*YKT6*		Same as above.
*GOSR2*	golgi SNAP receptor complex member 2	Encodes a trafficking membrane protein which transports proteins among the medial- and trans-Golgicompartments. Due to its chromosomal location and trafficking function, this gene may be involved in familial essential hypertension.

#### Calcium Signaling (CS) Pathway

The CS pathway was strongly activated in all three phases suggesting MAP infection has influence on this process during invasion and possibly related to MAP survival long term. Calcium signaling plays an important role in a broad range of regulatory effects on enzymes and proteins and influence on other major pathways including MAPK Signaling, Apoptosis, Long-term Potentiation, Long-term Depression, Phosphatidylinositol Signaling and others. Across all three immune phases, there were three significantly up-regulated genes that include *NFATC4*, *CAMK2A*, and *PLCB3* while there were another four genes that are significantly up-regulated only in the Early Phase that include *ADCY8*, *EDNRB*, *NFKBIB*, and *TACR2*. There were four significantly down-regulated genes that included *CALM2*, *PPID*, *GNAQ*, and *ATP2B4*. The biological roles of these genes are described in Table 12. NFATC4 plays a role in the inducible expression of cytokine genes in T cells, especially in the induction of the IL-2 and IL-4. However, there was no evidence of IL-2 or IL-4 expression in any phase of the host immune response of our study.

**Table 12:** Mechanistic Genes of the Calcium Signaling Pathway.

**Up-Regulated Mechanistic Genes in All Three Immune Response Phases**		
*NFATC4*	nuclear factor of activated T-cells, cytoplasmic, calcineurin-dependent 4	Encodes a protein that is member of the nuclear factors of activated T cells DNA-binding transcription complex that plays a role in the inducible expression of cytokine genes in T cells, especially in the induction of the IL-2 and IL-4.
*CAMK2A*	calcium/calmodulin-dependent protein kinase II alpha	Encodes protein that belongs to the serine/threonine protein kinases family, and to the Ca(2+)/calmodulin-dependent protein kinases (CaMKs) subfamily. CaMKs have numerous cellular functions and they influence processes as diverse as gene transcription, cell survival, apoptosis, and cytoskeletal re-organization.
*PLCB3*	phospholipase C, beta 3 (phosphatidylinositol-specific	Encodes a member of the phosphoinositide phospholipase C beta enzyme family that catalyze the production of the secondary messengers diacylglycerol and inositol 1,4,5-triphosphate from phosphatidylinositol in G-protein-linked receptor-mediated signal transduction. Phospholipases are ubiquitously expressed and have diverse biological functions including roles in inflammation, cell growth, signaling and death and maintenance of membrane phospholipids.
**Early Phase Only Up-Regulated Mechanistic Genes**		
*ADCY8*	adenylate cyclase 8	Encodes a protein that is a membrane bound enzyme that catalyses the formation of cyclic AMP from ATP
*EDNRB*	endothelin receptor type B	Encodes a G protein-coupled receptor which activates a phosphatidylinositol-calcium second messenger system.
*NFKBIB*	nuclear factor of kappa light polypeptide gene enhancer in B-cells inhibitor, beta	Encodes a protein that Inhibits NF-kappa-B by complexing with and trapping it in the cytoplasm. However, the unphosphorylated form resynthesized after cell stimulation is able to bind NF-kappa-B allowing its transport to the nucleus and protecting it to further NFKBIA-dependent inactivation.
*TACR2*	tachykinin receptor 2	Encodes the receptor for the tachykinin neuropeptide substance K, also referred to as neurokinin A (NK). This neurokinin 2 (NK2) receptor is a member of the tachykinin family of G-protein-coupled receptors. The NK2 receptor is predominantly expressed in the periphery gastrointestinal smooth muscle). NK2 receptors are thought to mediate gastrointestinal contraction and airway and lung function.
**Early Phase Only Down-Regulated Mechanistic Genes**		
*CALM2*	calmodulin 2 (phosphorylase kinase, delta)	Mediates the control of a large number of enzymes and other proteins by Ca(2+) and is involved in a genetic pathway that regulates the centrosome cycle and progression through cytokinesis.
*PPID*	peptidylprolyl isomerase D	Encodes a member of the peptidyl-prolylcis-trans isomerase (PPIase) family. PPIases catalyze the cis-trans isomerization of proline imidic peptide bonds in oligopeptides and accelerate the folding of proteins.
*GNAQ*	guanine nucleotide binding protein (G protein), q polypeptide	Encodes the guanine nucleotide binding protein that couples a seven-transmembrane domain receptor to activation of phospolipase C-beta.
*ATP2B4*	ATPase, Ca++ transporting, plasma membrane 4	Encodes a protein belonging to the family of P-type primary ion transport ATPases characterized by the formation of an aspartyl phosphate intermediate during the reaction cycle. These enzymes remove bivalent calcium ions from eukaryotic cells against very large concentration gradients and play a critical role in intracellular calcium homeostasis.

#### Host Immune Tolerance Subversion of Activated Immune Related Pathways

Defective sensing and killing of bacteria may drive the onset of chronic diseases like Johne's and Crohn's [49]. Although there are signs that the host is sensing the presence of MAP by generating an immune response in all phases, MAP successfully invades and evades the host immune processes. More specifically, the major immune related pathways that were activated in the Early Phase included the Toll-like Receptor Signaling, Hematopoietic Cell Linage, Adipocytokine Signaling Pathway, CD40L Signaling, Wnt Signaling, Cytokine-Cytokine Receptor Interactions, Complement and Coagulation Cascades, and Lectin Induced Complement pathways. Further examination of several of these pathways at the network level provided evidence that MAP was potentially interfering with their immune response functionalities.

#### Toll-like ReceptorSignaling (TLRS) Pathway Subversion

**Figure 6:**
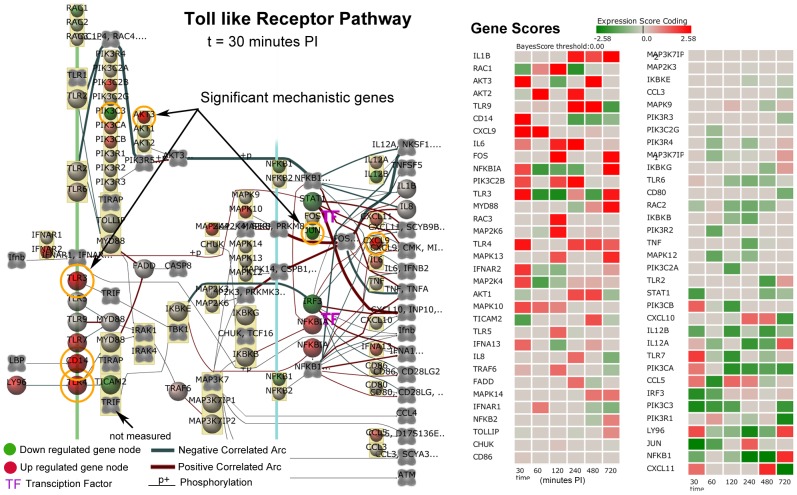
Toll-like Receptor Signaling Pathway. Toll-like receptor signaling pathway Bayesian network representation at 30 min post-infection (left), and the DBGGA scores for the gene expression of MAP infected host Peyer's patch versus non-infected controls (right). Gene nodes with orange circles on the network are those defined as mechanistic genes that surpass a threshold |Bayesian z-score|>2.24. The network shows gene nodes with gradient colors representing the level of expression (deeper red for higher up-regulated genes and deeper green for down-regulated). The heat map is colorized and corresponds to the gene node expression levels. Grey color represents little to no expression difference between MAP infected and controls. The heat map columns are by time post-infection in minutes.

With the triggering of the TLRS pathway, it could be presumed that the host had initiated an effective immune response. Examining this pathway at the network and gene expression level indicates that the source of pathway perturbation comes from genes that are both highly up-regulated and down-regulated over the complete time course. Figure 6 shows the Bayesian network for TLRS pathway and the Bayesian *z*-score gene expression temporal heat map for all genes on this pathway. The toll-like receptor signaling appears defective in that it is not producing the expected expression patterns for proinflammatory cytokines. The key cytokines, *IL-1β*, *TNF*, *IL-6*, and *IL-12* are not significantly expressed, although *IL-1β* is eventually up-regulated in the Late Phase. Also of interest are the chemokines *CCL3* (MIP-1α), *CCL5*(RANTES), *CXCL9*, *CXCL10*, and *CXCL11* which are not significantly expressed and suggests a potential disruption of monocyte and natural killer cell stimulation and T-cell migration that could explain, in part, the host immune tolerance for MAP. In the early and intermediate phase of MAP invasion, there is significant expression of *TLR4*, *TLR3* and *TLR9*, but no *TLR2* at any phase. TLR4 is expressed on the cell surface of enterocytes and numerous cells of the immune system such as dendritic cells, B lymphocytes and NK cells. On the other hand, MAP can also interact with *TLR9* located within the endosomal compartments of phagocytic cells and B lymphocytes and functions to alert the immune system of MAP infections. The lack of *TLR2* expression appears contrary to published results for *in vitro M. paratuberculosis* infected murine macrophages in which it was concluded that *TLR2* is one of the key recognition receptors [50], [51]. This could imply that the *in vivo* pathogenesis of MAP has differing invasion mechanism than *in vitro*, or the mechanisms are different between host species. Also there is no significant expression for *MYD88* or *NFkβ1* until the late phase in which *NFkβ1* eventually becomes significantly expressed.

#### Hematopoietic Cell Lineage (HCL) Pathway Subversion

The activation of the HCL pathway may also be an indicator of host immune response to MAP. The key genes that dominate the activation of HCL pathway are *IL-4R*, *CD14*, *CD59*, *GYPA*, *FLT3* and *CSF1R*. The biological roles of these genes are described in Table 13. Interleukin-4R is a receptor for both *IL-4* and *IL-13* and couples to the JAK1/2/3-STAT6 pathway. The *IL-4* response is involved in promoting Th2 cell differentiation. *CD14* is a surface antigen that is preferentially expressed on monocytes/macrophages. It cooperates with other proteins to mediate the innate immune response to bacterial lipopolysaccharide. *CD59* regulates complement-mediated cell lysis, and it is involved in lymphocyte signal transduction and is a potent inhibitor of the complement membrane attack complex while also playing a role in signal transduction pathways in the activation of T cells. *GYPA* is a major sialoglycoprotein of the erythrocyte membrane. Interestingly this protein has been linked to receptor-ligand interactions involved in the invasion of erythrocytes by malarial parasite [52] and may suggest a similar MAP influence. *FLT3* and its ligand *FLT3LG* play an important role in the immune response by regulating the functions of granulocytes/macrophage. As observed in our study, the *FLT3LG* gene expression was significantly down-regulated in the early phase and then up-regulated in the late phase. Inhibition of *FLT3LG* has been shown to significantly impair the immune system, as well as cause a reduction in myeloid progenitor cells. The number of B-cell progenitors, dendritic cells and natural killer cells have been reported to be significantly reduced in *in vivo* murine studies [53]. This suggests another evasion mechanism of MAP during the Early Phase that subverts the host immune response. *CSF1R* is the receptor for colony stimulating factor 1, a cytokine that controls the production, differentiation, and function of macrophages. This gene was up-regulated only at 30 minutes post-infection and was down-regulated in the intermediate and late phase suggesting a longer term mechanism of host immune tolerance to MAP. Our study clearly indicates that the host responses to MAP starts immediately after sensing the microbial interaction with the intestinal mucosa that in turn normally releases signals to stimulate recruitment of pro-inflammatory leucocytes, immune cells, or both.

**Table 13:** Mechanistic Genes for the Hematopoietic Cell Lineage (HCL) Pathway.

*IL-4R*	interleukin 4 receptor	Encodes the alpha chain of the interleukin-4 receptor, a type I transmembrane protein that can bind interleukin 4 and interleukin 13 to regulate IgE production. The encoded protein also can bind interleukin 4 to promote differentiation of Th2 cells. Receptor for both interleukin 4 and interleukin 13. Couples to the JAK1/2/3-STAT6 pathway. The IL4 response is involved in promoting Th2 differentiation. The IL4/IL13 responses are involved in regulating IgE production and, chemokine and mucus production at sites of allergic inflammmation.
*CD14*	CD14 molecule	Encodes a surface antigen that is preferentially expressed on monocytes/macrophages. It cooperates with other proteins to mediate the innate immune response to bacterial lipopolysaccharide. Cooperates with MD-2 and TLR4 to mediate the innate immune response to bacterial lipopolysaccharide (LPS). Acts via MyD88, TIRAP and TRAF6, leading to NF-kappa-B activation, cytokine secretion and the inflammatory response.Up-regulates cell surface molecules, including adhesion molecules.
*CD59*	CD59 molecule, complement regulatory protein	Encodes a cell surface glycoprotein that regulates complement-mediated cell lysis, and it is involved in lymphocyte signal transduction. This protein is a potent inhibitor of the complement membrane attack complex, whereby it binds complement C8 and/or C9 during the assembly of this complex, thereby inhibiting the incorporation of multiple copies of C9 into the complex, which is necessary for osmolytic pore formation. This protein also plays a role in signal transduction pathways in the activation of T cells. Involved in signal transduction for T-cell activation complexed to a protein tyrosine kinase
*GYPA*	glycophorin A (MNS blood group)	Encodes a major sialoglycoproteins of the human erythrocyte membrane which bear the antigenic determinants for the MN and Ss blood groups.
*FLT3*	fms-related tyrosine kinase 3	This gene encodes a class III receptor tyrosine kinase that regulates hematopoiesis. The receptor consists of an extracellular domain composed of five immunoglobulin-like domains, one transmembrane region, and a cytoplasmic kinase domain split into two parts by a kinase-insert domain. The activated receptor kinase subsequently phosphorylates and activates multiple cytoplasmic effector molecules in pathways involved in apoptosis, proliferation, and differentiation of hematopoietic cells in bone marrow.
*CSF1R*	colony stimulating factor 1 receptor	Encodes the receptor for colony stimulating factor 1, a cytokine which controls the production, differentiation, and function of macrophages. This receptor mediates most if not all of the biological effects of this cytokine.
*FLT3LG*	fms-related tyrosine kinase 3 ligand	Dendritic cells (DCs) provide the key link between innate and adaptive immunity by recognizing pathogens and priming pathogen-specific immune responses. FLT3LG controls the development of DCs and is particularly important for plasmacytoid DCs and CD8. Stimulates the proliferation of early hematopoietic cells by activating FLT3.

#### CD40L Signaling Pathway Subversion

Interaction between CD40L on activated T-cells and CD40 receptors on macrophages is crucial for maintaining a Th1 response and activation of macrophages [54]. The CD40L (ligand) signaling pathway was activated in the Early Phase, tended to be suppressed in the Intermediate Phase and strongly activated in the Late Stage as shown in Figure 1. CD40 relies on interaction with TRAF proteins to mediate an intracellular signal in response to CD40L binding. The pathway downstream of TRAFs activates the transcription factor NF-κB through 3 different kinase pathways involving MAP-kinases, NIK (NF-κB inducing kinase) and I-κB kinases [55]. All three of these kinases were down-regulated during MAP infection. Thus, during MAP infection, the antigen receptors of T-cells were stimulated; however, due to the lack of co-stimulator molecules from APCs, further T-cell activation apparently was greatly reduced, reducing the host response of immune activation to a level approaching an anergic state at the level of the intestine and influence the disease progression from paucibacillary form to the multibacillary form of the disease.

#### Cytokine-Cytokine Receptor Interactions (CCRI) Pathway Activation

The CCRI pathway was strongly activated in all three phases with only a few receptors dominating the activation. These genes were involved in extracellular membrane receptor interaction that included chemokines (CC and CXC), interleukins (ILs), platelet-derived growth factors (PDGFs), and tumor necrosis factors (TNFs). Chemokines and their receptors are important for the migration of various cell types into inflammatory sites. Only the genes *CCR4*, *CXCL9*, *BLR1* and *CCR8* were highly up-regulated in the Early Phase, while the remaining chemokines where moderately down-regulated or not expressed in this Phase. In the Intermediate and Late Phases, the previous chemokines were not expressed and the following chemokines become strongly up-regulated: *CCL24*, *CX3CL1*, *CCL8*, and *CCL20* while CXCL11 become strongly down-regulated. *CCR4* is a receptor for *CCL5*, and *CXCL11* is chemotactic for activated T cells. *BLR1* also known as CXCR5, has a role in Peyer's patch primary follicles relating to B cell migration [56]. The biological roles of these genes are described in Table 14. Studies of the CCL8 receptor and its ligands suggested its role in regulation of monocyte chemotaxis and thymic cell apoptosis. More specifically, this receptor may contribute to the proper positioning of activated T cells within the antigenic challenge sites and specialized areas of lymphoid tissues [57]. The gene *CCL20* may be involved in formation and function of the mucosal lymphoid tissues by attracting lymphocytes and dendritic cells towards epithelial cells.

**Table 14:** Chemokine Mechanistic Genes of the Cytokine-Cytokine Receptor Interactions Pathway.

**Early Phase Only Up-Regulated Mechanistic Genes**		
*CCR4*	chemokine (C-C motif) receptor 4	Encodes G-protein-coupled receptor family. It is a receptor for the CC chemokine - MIP-1, RANTES, TARC and MCP-1.
*CXCL9*	chemokine (C-X-C motif) ligand 9	The function of this gene has not been specifically defined; however, it is thought to be involved in T cell trafficking.
*BLR1*	chemokine receptor 5	Encodes a predicted seven transmembrane G protein- coupled receptor and belongs to the CXC chemokine receptor family, a BLC (B-lymphocyte chemoattractant). Cytokine receptor that binds to B-lymphocyte chemoattractant (BLC). Involved in B-cell migration into B-cell follicles of spleen and Peyer patches but not into those of mesenteric or peripheral lymph nodes. May have a regulatory function in Burkitt lymphoma (BL) lymphomagenesis and/or B-cell differentiation.
*CCR8*	chemokine (C-C motif) receptor 8	Encodes a member of the beta chemokine receptor family, which is predicted to be a seven transmembrane protein similar to G protein-coupled receptors.
**Intermediate and Late Phase Only Up-Regulated Mechanistic Genes**		
*CCL24*	chemokine (C-C motif) ligand 241	Encodes for a cytokine family of secreted proteins involved in immunoregulatory and inflammatory processes. Chemotactic for resting T-lymphocytes, and eosinophils. Has lower chemotactic activity for neutrophils but none for monocytes and activated lymphocytes. Is a strong suppressor of colony formation by a multipotential hematopoietic progenitor cell line.
*CX3CL1*	chemokine (C-X3-C motif) ligand 1	The soluble form is chemotactic for T-cells and monocytes, but not for neutrophils. The membrane-bound form promotes adhesion of those leukocytes to endothelial cells. May play a role in regulating leukocyte adhesion and migration processes at the endothelium. Binds to CX3CR1
*CCL8*	chemokine (C-C motif) ligand 8	Encodes a protein structurally related to the CXC subfamily of cytokines. This cytokine displays chemotactic activity for monocytes, lymphocytes, basophils and eosinophils. By recruiting leukocytes to sites of inflammation this cytokine may contribute to tumor-associated leukocyte infiltration and to the antiviral state against HIV infection.
*CCL20*	chemokine (C-C motif) ligand 20	Chemotactic factor that attracts lymphocytes and, slightly, neutrophils, but not monocytes. Inhibits proliferation of myeloid progenitors in colony formation assays. May be involved in formation and function of the mucosal lymphoid tissues by attracting lymphocytes and dendritic cells towards epithelial cells.
CXCL11	chemokine (C-X-C motif) ligand 11	Encodes a protein that induces a chemotactic response in activated T-cells and is the dominant ligand for CXC receptor-3. IFN-gamma is a potent inducer of transcription of this gene. Chemotactic for interleukin-activated T-cells but not unstimulated T-cells, neutrophils or monocytes. Induces calcium release in activated T-cells.

The function of the immune system depends in a large part on Interleukins that are predominately synthesized by helper CD4+ T lymphocytes, as well as through monocytes, macrophages, and endothelial cells. Interleukins promote the development and differentiation of T, B, and hematopoietic cells. The strongly expressed interleukins in the Early Phase include *IL-4R*, *IL-18RAP* and *IL-17RB*. IL-4R reverses expression direction and was strongly down regulated in the Intermediate and Late Phase. The strongly up-regulated ILs in the Intermediate and Late Phase include *IL-1B*, *IL-18RAP*, *IL-7*, *IL-8RB*, and *IL-6* while the strongly down-regulated genes include *IL-13*, *IL-15*, *IL-1A*, and *IL-4R*. The biological roles of these genes are described in Table 15. The soluble epithelial factors (*IL-7 and IL-15*) differentially regulate homeostasis of intraepithelial lymphocytes and other mucosal leukocytes. IL7 can be produced locally by intestinal epithelial and epithelial goblet cells, and may serve as a regulatory factor for intestinal mucosal lymphocytes. The IL8RB is a receptor for IL8 and mediates neutrophil migration to sites of inflammation. The angiogenic effects of IL8 in intestinal microvascular endothelial cells are mediated by this receptor. Leukocytes play an important role in the maintenance of epithelial barrier. Interestingly, the gene, *IL-13,* is known to be critical in regulating immune response, but it was strongly down-regulated in the host response and may be important to MAP survival long term.

**Table 15:** Interleukin Mechanistic Genes Cytokine-Cytokine Receptor Interactions Pathway.

**Early Phase Only Up-Regulated Mechanistic Genes**		
*IL-4R*	interleukin 4 receptor	Encodes the alpha chain of the interleukin-4 receptor, a type I transmembrane protein that can bind interleukin 4 and interleukin 13 to regulate IgE production.
*IL-18*	interleukin 18(interferon-gamma-inducing factor)),	Encodes a proinflammatory cytokine. This cytokine can induce the IFN-gamma production of T cells. The combination of this cytokine and IL12 has been shown to inhibit IL4 dependent IgE and IgG1 production, and enhance IgG2a production of B cells. IL-18 binding protein (IL18BP) can specifically interact with this cytokine, and thus negatively regulate its biological activity.
*IL-18RAP*	interleukin 18 receptor accessory protein	Encodes an accessory subunit of the heterodimeric receptor for IL18. This protein enhances the IL18 binding activity of IL18R1 (IL1RRP), a ligand binding subunit of IL18 receptor. Required for the high affinity binding of interleukin 18 (IL-18) to its receptor complex (By similarity). Together with IL18R1 mediates IL-18-dependent activation of NF-kappa-B and JNK
*IL-17RB*	interleukin receptor B	Encodeds a cytokine receptor. This receptor specifically binds to IL17B and IL17E, but does not bind to IL17 and IL17C. This receptor has been shown to mediate the activation of NF-kappaB and the production of IL8 induced by IL17E.
**Intermediate and Late Phase Only Up-Regulated Mechanistic Genes**		
*IL-1B*	interleukin 1, beta	Encodes a member of the interleukin 1 cytokine family. This cytokine is produced by activated macrophages as a proprotein, which is proteolytically processed to its active form by caspase 1 (CASP1/ICE). This cytokine is an important mediator of the inflammatory response, and is involved in a variety of cellular activities, including cell proliferation, differentiation, and apoptosis.
*IL-18RAP*	See description above	
*IL-7*	interleukin 7	Encodes a cytokine important for B and T cell development. This cytokine and the hepatocyte growth factor (HGF) form a heterodimer that functions as a pre-pro-B cell growth-stimulating factor. This cytokine is found to be a cofactor for V(D)J rearrangement of the T cell receptor beta (TCRB) during early T cell development. This cytokine can be produced locally by intestinal epithelial and epithelial goblet cells, and may serve as a regulatory factor for intestinal mucosal lymphocytes.
*IL-8RB*	chemokine (C-X-C motif) receptor 2	Encodes a member of the G-protein-coupled receptor family. This protein is a receptor for interleukin 8 (IL8). It binds to IL8 with high affinity, and transduces the signal through a G-protein activated second messenger system. This receptor mediates neutrophil migration to sites of inflammation. The angiogenic effects of IL8 in intestinal microvascular endothelial cells are found to be mediated by this receptor.
*IL-6*	interleukin 6 (interferon, beta 2)	Encodes a cytokine that functions in inflammation and the maturation of B cells. It is a potent inducer of the acute phase response. Plays an essential role in the final differentiation of B-cells into Ig-secreting cells Involved in lymphocyte and monocyte differentiation.
**Intermediate and Late Phase Only Down-Regulated Mechanistic Genes**		
*IL-13*	interleukin 13	Encodes an immunoregulatory cytokine produced primarily by activated Th2 cells. This cytokine is involved in several stages of B-cell maturation and differentiation. Inhibits inflammatory cytokine production. Synergizes with IL2 in regulating interferon-gamma synthesis. May be critical in regulating inflammatory and immune responses.
*IL-15*	interleukin 15	Encodes a cytokine that regulates T and natural killer cell activation and proliferation.
*IL-1A*	interleukin 1, alpha	Encodes a member of the interleukin 1 cytokine family. This cytokine is a pleiotropic cytokine involved in various immune responses, inflammatory processes, and hematopoiesis. This cytokine is produced by monocytes and macrophages as a proprotein, which is proteolytically processed and released in response to cell injury, and thus induces apoptosis.
*IL-4R*	See description above	

The elevated expression of platelet-derived growth factors (PDGF) have been linked to early signaling events for infection by intracellular pathogens [58]. PDGF genes and their receptors that were strongly up-regulated in the Early Phase include *VEGFB*, *FLT3*, *FLT3LG*, and *CSF1R*. *VEGFB* signals via the endothelial receptor FLT1 and is a regulator of blood vessel physiology, with a role in endothelial targeting of lipids to peripheral tissues. *FLT3* encodes a class III receptor tyrosine kinase that regulates hematopoiesis. *CSF1R* encodes a tyrosine kinase transmembrane receptor and is involved in the functions of macrophages. Expression levels subsided for *VEGFB*, *FLT2LG*, and *CSF1R* in the Intermediate and Late Phase, but *FLT3* was triphasic, in that it was highly up-regulated in the Early Phase, highly down-regulated in the Intermediate Phase and becomes highly up-regulated in the Late Phase. Only *FLT1* and *VEGFC* were up-regulated in the Intermediate and Late Phases while *KDR* and *PDGFC* were strongly down-regulated. *FLT1* encodes a receptor tyrosine-kinase and plays a key role in vascular development and regulation of vascular permeability. *VEGFC* encodes a PDGF that has a role in endothelial cell growth, stimulating their proliferation and migration and also has effects on the permeability of blood vessels. The down-regulated *KDR* gene encodes one of the two receptors of the VEGF and is a main mediator of VEGF-induced endothelial proliferation, survival, migration, tubular morphogenesis and sprouting. The down-regulated gene *PDGFC* is a receptor with tyrosine-kinase activity that has roles in the regulation of many biological processes including embryonic development, angiogenesis, cell proliferation and differentiation, and contribute to the pathophysiology of some diseases, including cancer. Biological roles of these genes are provided in Table 16.

**Table 16:** Platelet Derived Growth Factors Mechanistic Genes of the Cytokine-Cytokine Interaction Pathway.

**Early Phase Only Up-Regulated Mechanistic Genes**		
*VEGFB*	vascular endothelial growth factor B	Vascular endothelial growth factor B (VEGFB) signals via the endothelial receptor VEGFR1 (MIM 165070) and is a regulator of blood vessel physiology, with a role in endothelial targeting of lipids to peripheral tissues
*FLT3*	fms-related tyrosine kinase 3**^1^**	Encodes a class III receptor tyrosine kinase that regulates hematopoiesis. The receptor is activated by binding of the fms-related tyrosine kinase 3 ligand to the extracellular domain, which induces homodimer formation in the plasma membrane leading to autophosphorylation of the receptor. The activated receptor kinase subsequently phosphorylates and activates multiple cytoplasmic effector molecules in pathways involved in apoptosis, proliferation, and differentiation of hematopoietic cells in bone marrow.
*FLT3LG*	fms-related tyrosine kinase 3 ligand	Stimulates the proliferation of early hematopoietic cells by activating FLT3. Synergizes well with a number of other colony stimulating factors and interleukins
*CSF1R*	colony stimulating factor 1 receptor	Encodes the receptor for colony stimulating factor 1, a cytokine which controls the production, differentiation, and function of macrophages. This receptor mediates most if not all of the biological effects of this cytokine. Ligand binding activates the receptor kinase through a process of oligomerization and transphosphorylation. The encoded protein is a tyrosine kinase transmembrane receptor and member of the CSF1/PDGF receptor family of tyrosine-protein kinases.
**Intermediate and Late Phase Only Up-Regulated Mechanistic Genes**		
*FLT1*	fms-related tyrosine kinase 1 (vascular endothelial growth factor/vascular permeability factor receptor)	Encodes a receptor tyrosine- kinase and plays a key role in vascular development and regulation of vascular permeability. Vascular endothelial growth factor is a signaling protein involved in the regulation of angiogenesis and vasculogenesis. VEGF binds to and activates a receptor tyrosine kinase, VEGFR.
*VEGFC*	vascular endothelial growth factor C	Encodes a PDGF that has a role in endothelial cell growth, stimulating their proliferation and migration and also has effects on the permeability of blood vessels.
**Intermediate and Late Phase Only Down-Regulated Mechanistic Genes**		
*KDR*	kinase insert domain receptor (a type III receptor tyrosine kinase))	Encodes one of the two receptors of the VEGF and is a main mediator of VEGF-induced endothelial proliferation, survival, migration, tubular morphogenesis and sprouting.
*PDGFC*	platelet derived growth factor C	Encodes a receptor with tyrosine-kinase activity that has roles in the regulation of many biological processes including embryonic development, angiogenesis, cell proliferation and differentiation, and contribute to the pathophysiology of some diseases, including cancer.

#### Adipocytokine Signaling (AS) Pathway Manipulation

The activated AS pathway may be a novel pathway associated with MAP invasion. Several adipocytokines have been found to have a central role in the regulation of inflammation and immunity [59] and may be important as another MAP host evasion strategy. Adipocytokines exert different effects on the innate immune system and either suppress or activate the monocyte–macrophage system. *ADIPOQ* (gene encoding adiponectin) through interaction with its receptor *ADIPOR1* (and *ADIPOR2*) suppresses the NF-kB-dependent synthesis of tumour-necrosis factor (TNF) and interferon (IFN). Adiponectin also induces apoptosis of monocytes and inhibits phagocytosis by macrophages.

#### Wnt Signaling (WS) Pathway Activation

In our study, Wnt signaling, which was a highly scored pathway during the Early Phase, is known to be associated with regeneration of nervous system cells using an integrative computational model for intestinal tissue renewal [60]. Planar cell polarity signaling is one of the downstream pathways in the Wnt signaling, and it leads to the activation of the small *GTPases* and *Rac-1*. *Rac-1*, one of the mechanistic genes identified in our study, may regulate cell adhesion and epithelial cell motility in response to MAP entry. In neurons, *Rac-1* acts through the *protein kinase cdk5* and *p35* to phosphorylate and down-regulate *Pak1*, increasing neuronal migration. *Rac-1* also interacts with several other factors to regulate enteric neuronal network. *Neurexophilins* (neuropeptide-like proteins) and *neurexins* also participate in a neuron signaling pathway [61]–[63]. *Neuronactin* and *contactin* transmembrane proteins are also known to mediate cell-cell interactions in nervous system [64]. *Neurexins* contain epidermal growth factor-like sequences and domains homologous to the G domain repeats of laminin A, as related to its function in ileal mucosa and cell-cell interactions. Intestinal motility is affected by the invading enteric pathogens. A number of gastrointestinal hormones appear to affect intestinal motility [65]. Interestingly, it was shown earlier that the enteric nervous system is involved in inflammatory bowel disease in which MAP is associated [66], [67]. In cattle and sheep with Johne's disease, myenteric ganglionitis with cellular infiltration occurs [68]. During the experimental inoculation of sheep with MAP, some sheep developed aggregations of mononuclear cells around enteric nerves in the ileal submucosa and myenteric plexus [69]. However, such lesions were not detected in sheep that did not subsequently develop classical disease manifestations. At this juncture, it is not clear if the Johne's disease is also an outcome of the enteric neuropathy that starts when MAP colonizes in the intestine. Thus, further studies are warranted to understand the correlation between MAP colonization and enteric neuropathy. Furthermore, *neurotrophins* are known to activate two different classes of receptors, the Trk family of receptor tyrosine kinases and p75NTR, a member of the TNF receptor superfamily. Our gene expression data indicate that neurotrophins may be activating TNF receptor superfamily. This interaction may in turn activate many signaling pathways, including those mediated by *ras* and members of the *cdc-42/ras/rho G protein* families, and by the *MAP kinase*, *PI-3 kinase*, *and Jun kinase* cascades.

#### Mitogen-activated Protein Kinase 1 (MAPK1) Influence on MAP invasion

**Figure 7:**
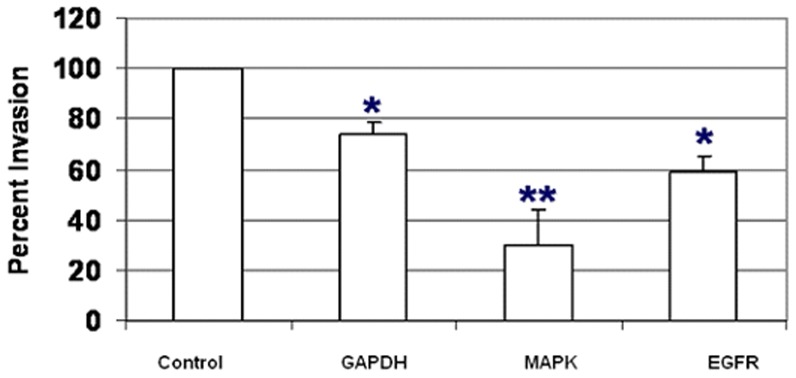
Effect of siRNA Knock-down of MAPK1 on the Invasion of HeLa cells by MAP. HeLa cells were transfected with Glyceraldehyde 3 phosphate dehydrogenase (GAPDH), mitogen activated protein kinase 1 (MAPK1), and Epithelial Growth Factor Receptor (EGFR) siRNAs, infected with MAP, and measured for the effects on invasion. Invasion is given as a percentage relative to the negative control that consisted of HeLa cells incubated with transfecting reagent in serum free medium.

In our study we focused on the MAPK1 gene. The protein encoded by *MAPK1* is a member of the MAP kinase family. MAP kinases, also known as extracellular signal-regulated kinases (ERKs), act as an integration point for multiple biochemical signals, and are involved in a wide variety of cellular processes such as proliferation, differentiation, transcription regulation and development. Unlike other pathogens, MAP did not exhibit increased invasion or replication during the 12 hr post-infection, though MAP was in continuous contact with host Peyer's patch in the ligated ileal loop [2]. To provide evidence that inhibition in the entry of MAP is MAPK1 dependent, we specifically knocked down *in vitro* MAPK1 gene expression in HeLa cells by siRNA. The invasion of MAP in HeLa cells was highly significantly reduced when we silenced *MAP kinase* by introducing siRNA (Figure 7). Thus, *MAP kinase* is probably one of the key genes influencing invasion of MAP.

### Host Immune Tolerance: Suppressed Immune Related Pathways

Pathways that are suppressed may be assumed to be an indicator of MAP host processes that are hi-jacked, but in a way to subvert the host's defensive response. The One Carbon Pool by Folate, Long-term Potentiation, Long-term Depression, and CCR3 Signaling in Eosinophils pathways are potentially hi-jacked processes suppressed in the Early Phase. Further examination of these pathways at the network level provided evidence that MAP was potentially interfering with their immune response functionality.

#### One Carbon Pool By Folate (OCPF) Pathway Suppression

The OCPF pathway is suppressed in the Early Phase, was tri-phasic (suppressed, activated, and suppressed) in the Intermediate Phase and was strongly activated in the Late Phase as shown in the pathway heat map of Figure 1. This pathway may be novel to MAP pathogenicity and its impairment may adversely impact genome integrity, disrupt establishment of other metabolic pathways and mechanisms that underlie folate-associated pathologies. A previous study [70] found that folate deficiency inhibits the proliferation of primary CD8+ T Lymphocytes, another possible mechanism underlying host immune tolerance of MAP. Within the One Carbon Pool by Folate pathway are three strongly down-regulated genes (Early Phase) that include *MTHFD1*, *MTHFD2*, and *GART*. In the Late phase, *GART* reversed to become strongly up-regulated while *MTHFD1* and *MTHFD2* reduced expression levels to a moderate insignificant level. A review of the literature found *MTHFD1* and *MTHFD2* to have some association with immune response. The *MTHFD1* encodes a protein that possesses three distinct enzymatic activities that are essential cofactors for thymidylate and purine synthesis [71]. Disorders of purine metabolism lead to immunodeficiency having marked susceptibility to infection [72]. Interestingly, the protein encoded by *MTHFD2* was found to have highly fluctuating protein abundance levels over time in mouse macrophages infected with *Salmonella enterica* [73]. This suggests that *MTHFD1* and *MTHFD2* may be novel to the MAP invasion mechanisms and may warrant further examination in future studies. More details of the biological role of these genes are provided in Table 17.

**Table 17:** Strongly Down-Regulated Mechanistic Genes of the One Carbon Pool by Folate Pathway.

*MTHFD1*	methylenetetrahydrofolate dehydrogenase (NADP+ dependent) 1, methenyltetrahydrofolate cyclohydrolase, formyltetrahydrofolate synthetase1	This gene encodes a protein that possesses three distinct enzymatic activities, 5,10-methylenetetrahydrofolate dehydrogenase, 5,10-methenyltetrahydrofolate cyclohydrolase and 10-formyltetrahydrofolate synthetase.
*MTHFD2*	methylenetetrahydrofolate dehydrogenase (NADP+ dependent) 2, methenyltetrahydrofolate cyclohydrolase	This gene encodes a nuclear-encoded mitochondrial bifunctional enzyme with methylenetetrahydrofolate dehydrogenase and methenyltetrahydrofolate cyclohydrolase activities. The enzyme functions as a homodimer and is unique in its absolute requirement for magnesium and inorganic phosphate.
*GART*	phosphoribosylglycinamide formyltransferase, phosphoribosylglycinamide synthetase, phosphoribosylaminoimidazole synthetase	Encodes a trifunctional polypeptide. It has phosphoribosylglycinamide formyltransferase, phosphoribosylglycinamide synthetase, phosphoribosylaminoimidazole synthetase activity which is required for de novo purine biosynthesis.

#### Long-term Potentiation (LTP) and Long-term Depression (LTD) Pathway Suppressions

Other novel pathways suppressed in the early phase and reversed to an activated state in the late phase are the LTP and LTD pathways. MAP pathogenicity appears to have interaction with neuronal activity, the mechanisms of which are not well understood. The dominating genes causing the pathways' suppressed scores are *PPP1CA*, *PPP1CB*, *MAPK1*, *GNAI3*, *GNAO1*, *IGFR1*, and *Gucy2c*. The biological roles of these genes are provided in Table 18. Recently, it was found that Gucy2c is involved in regulating AKT-dependent intestinal barrier integrity [74]. *GNAI3* has been linked as an important participant in lymphocyte position and chemokine receptor signaling in B cells [75].

**Table 18:** Key Mechanistic Genes of the Long-term Potentiation and Long-term Depression Pathways.

**Early Phase Down-Regulated Genes**		
*PPP1CA*	protein phosphatase 1, catalytic subunit, alpha isozyme1	Encodes one of the three catalytic subunits of protein phosphatase 1 (PP1). PP1 is a serine/threonine specific protein phosphatase known to be involved in the regulation of a variety of cellular processes, such as cell division, glycogen metabolism, muscle contractility, protein synthesis, and HIV-1 viral transcription. Increased PP1 activity has been observed in the end stage of heart failure. Studies in both human and mice suggest that PP1 is an important regulator of cardiac function.
*PPP1CB*	protein phosphatase 1, catalytic subunit, beta isozyme1	Same as *PPP1CA* above
*MAPK1*	mitogen-activated protein kinase 11	Encodes a member of the MAP kinase family. MAP kinases, also known as extracellular signal-regulated kinases (ERKs), act as an integration point for multiple biochemical signals, and are involved in a wide variety of cellular processes such as proliferation, differentiation, transcription regulation and development. Involved in both the initiation and regulation of meiosis, mitosis, and postmitotic functions in differentiated cells by phosphorylating a number of transcription factors such as ELK1. Phosphorylates EIF4EBP1; required for initiation of translation.
*GNAI3*	guanine nucleotide binding protein (G protein), alpha inhibiting activity polypeptide 3	Guanine nucleotide-binding proteins (G proteins) are involved as modulators or transducers in various transmembrane signaling systems.
*GNAO1*	guanine nucleotide binding protein (G protein), alpha activating activity polypeptide O	Guanine nucleotide-binding proteins (G proteins) are involved as modulators or transducers in various transmembrane signaling systems. The G(o) protein function is not clear.
*IGFR1*	insulin-like growth factor 1 receptor1	This receptor binds insulin-like growth factor with a high affinity. It has tyrosine kinase activity. The insulin-like growth factor I receptor plays a critical role in transformation events. IGFRs mediate their intracellular actions through the PI 3-K and RAS/RAF/MAPK signaling pathways and downstream effectors include mTOR, p70 S6 kinase, ERK and JNK. Many tumors have altered expression of IGF1R and its ligands and this constitutes an early, possible initiating, event in tumorigenesis.
*Gucy2c*	guanylate cyclase 2C (heat stable enterotoxin receptor	Receptor for the E.coli heat-stable enterotoxin (E.coli enterotoxin markedly stimulates the accumulation of cGMP in mammalian cells expressing GC-C). Also activated by the endogenous peptide guanylin.

#### CCR3 Signaling in Eosinophils (CSE) Pathway Suppression

In our study, CSE pathway was strongly suppressed in the Early Phase, inactive in the Intermediate Phase, and moderately suppressed in the Late Phase. CSE pathway suppression may be a key mechanism that supports the host tolerance to MAP. Eosinophils are a key class of leukocytes involved in inflammatory responses. Blocking eosinophil activation and the signaling pathways that lead to chemotaxis, degranulation and reactive oxygen release may alleviate inflammatory conditions and inflammation-associated tissue damage which may be a longer term survival mechanism of MAP. A number of genes are strongly down-regulated or not differentially expressed at all during the Early Phase and the majority of genes are lowly expressed in the Intermediate and Late Phases. In fact, the gene *CCR3* has a low differential expression across all phases. The protein encoded by *CCR3* is a receptor for C-C type chemokines and belongs to family 1 of the G protein-coupled receptors. This receptor binds and responds to a variety of chemokines, including eotaxin (CCL11), eotaxin-3 (CCL26), MCP-3 (CCL7), MCP-4 (CCL13), and RANTES (CCL5). It is highly expressed in eosinophils and basophils, and is also detected in TH1 and TH2 cells. The key genes dominating the suppression of CCR3 Signaling in the Early Phase are *GNAQ*, *MAP2K1*, and *MYL2* although there are several other genes that had a moderate down regulation. Biological roles of these genes are provided in Table 19. *GNAQ* was described previously in the Calcium Signaling pathway. *MAP2K1* is an essential component of MAP kinase signal transduction pathway, involved in many cellular processes such as proliferation, differentiation, transcription regulation and development and be also be important for eosinophil chemotaxis. The gene *MYL2* encodes the myosin, light chain 2 protein. In CSE pathway, *MYL2* is associated with the assembly of actomysin filaments. It is also involved in muscle contraction through cyclic interactions with actin-rich thin filaments, creating acontractile force. CSE pathway suppression may disrupt immune defenses by alleviating inflammatory conditions. This may signify another contributing mechanism to host immune tolerance of MAP.

**Table 19:** Key Down-Regulated Mechanistic Genes of the CCR3 Signaling in Eosinophil Pathway.

*GNAQ*	guanine nucleotide binding protein (G protein), q polypeptide1	The encoded protein, an alpha subunit in the Gq class, couples a seven-transmembrane domain receptor to activation of phospolipase C-beta. Mutations at this locus have been associated with problems in platelet activation and aggregation.
*MAP2K1*	mitogen-activated protein kinase kinase 1	Encodes a member of the dual specificity protein kinase family, which acts as a mitogen-activated protein (MAP) kinase kinase. MAP kinases, also known as extracellular signal-regulated kinases (ERKs), act as an integration point for multiple biochemical signals. As an essential component of MAP kinase signal transduction pathway, this kinase is involved in many cellular processes such as proliferation, differentiation, transcription regulation and development.
*MYL2*	myosin, light chain 2, regulatory, cardiac, slow1	encodes the regulatory light chain associated with cardiac myosin beta (or slow) heavy chain. Ca+ triggers the phosphorylation of regulatory light chain that in turn triggers contraction. Myosins are a large family of motor proteins that share the common features of ATP hydrolysis, actin binding and potential for kinetic energy transduction.

During the Intermediate Phase of MAP infection, higher levels of perturbation of signaling and immune response related pathways reflect active host-pathogen interactions. Autocrine and paracrine cell-cell signaling are very important for the interaction and maintenance of homeostasis within the diverse cell population (enterocytes, dendritic cells, macrophages, eosinophils, mast cells, and natural killer cells) of the intestinal mucosa [76], [77].

### Host Immune Tolerance: Non-Responsive Immune Related Pathways

Pathways that are non-responsive (not activated) may be an indicator of disrupted host processes by MAP. An interesting finding was that interaction of MAP with the host failed to induce several key immune related pathways during all three phases of host response. These pathways included Fc Epsilon RI Signaling, B cell Receptor (BCR) Signaling, Activation of CSK Through T Cell Receptor Signaling, Natural Killer Cell Mediated Cytotoxicity, and T Cell Receptor Signaling. Fc epsilon RI signaling is exclusive to the mast cells [78]. Suppression of mast cells activity thus affect the innate responses of the host to release several activated molecules, such as biogenic amines (histamines), proteoglycans (heparin), lipid mediators such as leukotrienes (LTC4, LTD4 and LTE4), prostaglandins (especially PDG2) and secretion of cytokines, the most important of which are TNF-α, IL4 and IL5. The suppression of these mediators, cytokines and T-cell receptors signaling along with the up-regulation in the epithelial repair mechanisms and reduced inflammation may enhance MAP intracellular survival and facilitate persistent infection. BCR Signaling inactivity may imply that any signaling pathways emanating from the B cell antigens is likely not stimulating any B lymphocyte immune response.

**Figure 8:**
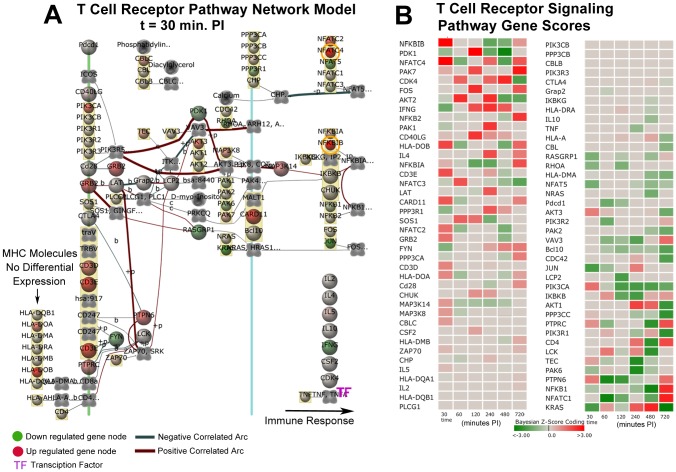
T-Cell Receptor Signaling Pathway Network Model and T-Cell Signaling Pathway Scores. The model (a) and the heat map (b) indicate an overall lack of differential expression that reflects a defective host immune response state to MAP in bovine Peyer's patch. A defective antigen processing and presentation by MHC class II molecule and lack of immune response is readily evident by examining the network (a) and the Bayesian *z-*score heat map (b).

To further understand the mechanistic events that are suppressing T-cell activation, the CD40L Signaling (CS) and T-cell Signaling (TCS) pathways were examined at the gene expression and network level. Table 20 indicates the DBGGA gene Bayesian z-score results across the Early, Intermediate and Late Phases of host immune response for CS pathway that shows a majority of important genes as not significantly expressed. Suppression of genes in the CS pathway may have a further negative regulation on a large number of genes implicated in host defense against pathogens. For the TCS pathway, the DBGGA analysis clearly indicated a defective antigen processing and presentation by MHC class II molecule as shown in Figure 8a. In this graphical representation of T-cell signaling, several genes encoding MHC molecules were not differentially expressed. MAP infection did not change the expression level of co-stimulatory molecules (*CD28*, *CD24*, *CD40LG and CD80*) that are known to be involved in the activation of *PI3K* and *GRB2* that finally activate *NFκB*. Interestingly, during MAP infection, *LCK* and *NFATC1* (nuclear factor of activated T-cells, cytoplasmic, calcineurin-dependent 1), *NFATC4* (nuclear factor of activated T-cells, cytoplasmic, calcineurin-dependent 4) are the mechanistic genes in T-cell signaling pathway and activation of these genes leads to ubiquitin-mediated proteolysis. The *NFATC1* and NFATC4 genes were common mechanistic genes in VEGF signaling pathway and T-cell receptor signaling pathway. NFATC1 was strongly down-regulated in the Early Phase and up-regulated in the Late Phase while NFATC4 was only strongly up-regulated in the Early Phase. The products of *NFATC1* and *NFATC4* genes play a role in the inducible expression of cytokine genes in T-cells, especially in the induction of the *IL-2* or *IL-4* gene transcription that, in our study, were not differentially expressed. These gene products are also involved in regulation, activation, proliferation and differentiation of T-cells as well as lymphoid and non-lymphoid cells. Several of these genes are involved in maintaining a fine balance between immunity and tolerance.

**Table 20:** Bayesian *z-*Score Values of Genes Involved in the Activation of CD40 Molecule.

Symbol	Description	t = 30	t = 60	t = 120	t = 240	t = 480	t = 720
TRAF6	TNF receptor-associated factor 6	1.01	0.07	1.65	0	0	−2.16
TRAF3	TNF receptor-associated factor 3	0	0	0	0	−0.76	0.92
TNFAIP3	Tumor necrosis factor, alpha-induced protein 3	−1.58	0	1.88	0	−0.54	−1.08
NFKBIA	Nuclear factor of kappa light polypeptide gene enhancer in B-cells inhibitor, alpha	0.79	−0.38	−1.71	−1.33	−0.26	2.12
NFKB1	Nuclear factor of kappa light polypeptide gene enhancer in B-cells 1 (p105)	−1.47	0	0	−0.59	−2.6	2.25
MAP3K14	Mitogen-activated protein kinase kinase kinase 14	1.31	−0.22	−0.65	−0.35	−0.45	0
MAP3K1	Mitogen-activated protein kinase kinase kinase 1	2.74	0	−2.83	−2.58	4.83	−2.24
IKBKG	Inhibitor of kappa light polypeptide gene enhancer in B-cells, kinase gamma	−0.14	0	0	0	−1.6	0.66
IKBKB	Inhibitor of kappa light polypeptide gene enhancer in B-cells, kinase beta	0	0.31	−0.43	−1.63	0	1.17
IKBKAP	Inhibitor of kappa light polypeptide gene enhancer in B-cells, kinase complex-associated protein	1.74	0	2.41	0.31	−0.37	0
DUSP1	Dual specificity phosphatase 1	1.69	0.65	1.81	0	0	2.4
CHUK	Conserved helix-loop-helix ubiquitous kinase	0	0	0.69	0.17	0	−0.66

t = time (in minutes) post-infection.

Moreover, the expression of all the genes related to MHC molecules (*HLA-DMA*, *HLA-A*, *HLA-DQB2*, *HLA-DRA*, *HLA-DQA1*, *HLA-DMB*, *HLA-DOA*, *HLA-DOB*) were not differentially expressed or tended to be down-regulated the entire period of our experiments (Figure 8b). Full T-cell activation requires: 1) binding of the T-cell receptor to the antigen-MHC complex on the antigen presenting cell, and 2) a co-stimulatory signal provided by the binding of the T-cell's CD28 protein to the B7 protein on the Antigen Presenting Cell (APC). In our study, MAP infection resulted in down-regulation of gene expression of MHC molecules at all the time points post-inoculation. Table 21 summarizes the responses or expression of TLR and MHC genes upon interaction of MAP with several host systems [79]–[86]. The MHC down-regulation has also been demonstrated to occur in *in vitro* grown macrophages (as early as 12 hr post-infection), as well as, in macrophages isolated from subclinical and clinical phase of infection [7], [87], [88]. Thus, it is plausible that the down-regulation of the MHC molecules observed in the present study at the Early Phase of infection initiates and facilitates permanently persistent MAP infections. Given the role of MHC molecules in triggering the Th cells, the down-regulation of MHC during the establishment of MAP infection may block the effector arms of immune system. This irreversible down-regulation of MHC expression may contribute at some level to the paucity of T-cell infiltrates and tubercle formation observed in Johne's disease lesions [89].

**Table 21:** Summary Review of MAP-Induced TLR and MHC Responses.

Infection Model	Expression level	Reference
Blood samples from four MAP exposed and four unexposed cattle	Unconfirmed association between MAP exposure and *in-vivo* MHC gene modulation	[83]
In silico analysis, for a population-based genetic association of a Spanish Holstein-Friesian sample	3 tightly linked SNP in TLR4 were found to be associated with susceptibility to MAP infection	[84]
Early paucibacillary MAP infection compared to multibacillary MAP infection in sheep	Lower expression levels of most TLRs in paucibacillary group than the multibacillary group	[85]
MAP infected monocyte-derived dendritic cells	Unable to present antigen via MHC class II	[82]
In vitro infection of Chinese Hamster Ovary (CHO) cells with MAP	Low TLR4	[81]
Peripheral blood derived macrophages from juvenile sheep	Down-regulation of MHC class I and II	[80]
Bovine macrophages infection (*in-vitro*) with MAP	Down-regulation of MHC class I and class II molecules	[86]
Intestinal macrophages in clinically infected sheep compared with normal tissues	Lower expression of MHC class II	[79]

### Other Mechanistic Genes of Interest

Expression levels of keratinocyte growth factor *(KGF*), insulin-like growth factor-1 (*IGF* secreted by intraepithelial lymphocytes) and macrophage-derived factors were highly up-regulated during the Late Phase of infection. Several genes that encode for antimicrobial mechanisms against any invading pathogens were down-regulated in the MAP infection. These included *nitric oxide synthase* (endothelial as well as hepatic), *dipepdidyl-peptidase*, and *dihydrofolate reductase (DHFR)* at all the time points. *DHFR* is critical for nitric oxide bioavailability in bovine aortic endothelium cells [90].

### Host Immune Tolerance by Antigen Mimicry

Immune tolerance can also be induced via antigen mimicry. In Crohn's patients, amino acid similarities between MAP and intestinal proteins was examined in detail [91]. Auto-reactive lymphocytes specific for glutathione peroxidase participate in the decreased activity of this enzyme observed in Crohn's disease patients. This in turn could lead to an imbalanced and inefficient endogenous antioxidant response in the intestinal mucosa of Crohn's disease patients. Further studies are warranted to understand if a similar kind of antigen mimicry occurs in Johne's disease.

### Biological System Level Modeling

**Figure 9:**
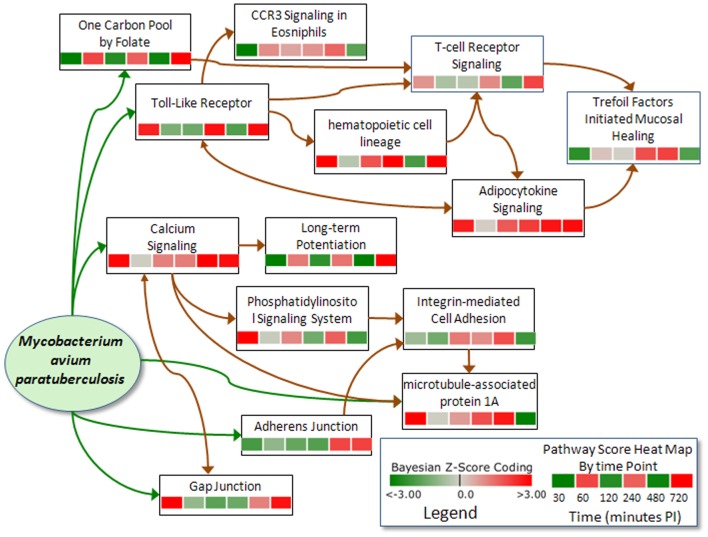
High Level Conceptual Systems Network of Interrelated Pathways further Defining the Bovine Host Immune Tolerance to MAP. Each box that defines specific pathways has its temporal heat map scores to document the dynamic state of the pathway (see figure legend). The arrows connecting the pathways are drawn to represent possible causal relationships between the pathways.

**Figure 10:**
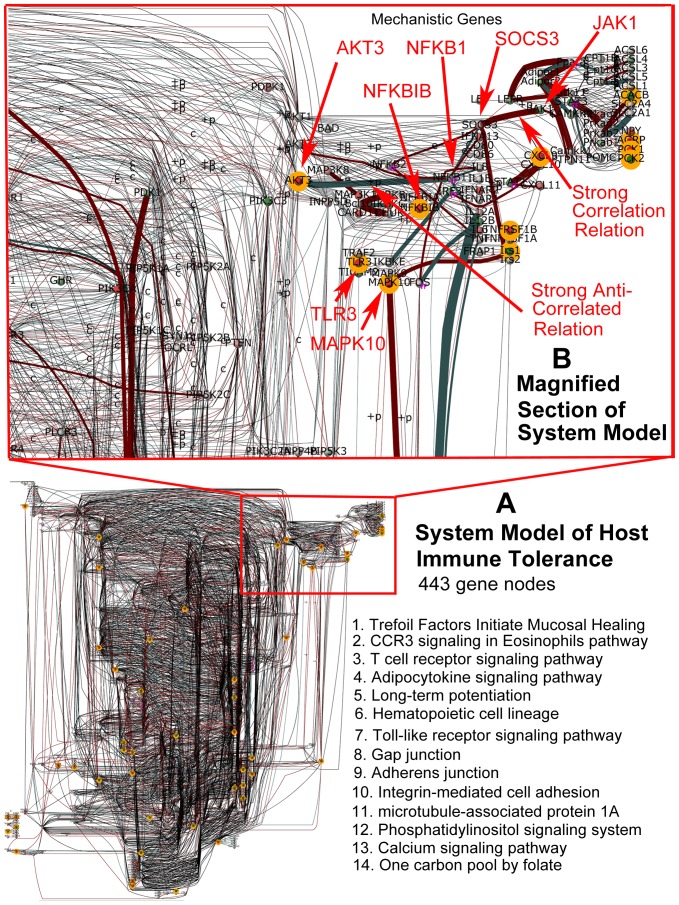
System Bayesian Network Model of Host Immune Tolerance. Fourteen pathways were used to construct the network developed from temporal *in vivo* host transcriptome data of MAP infected bovine Peyer's patch. The full network model is shown on the bottom figure. A magnified section of the model is shown in the top figure. Mechanistic genes are shown with orange rings. A few important mechanistic genes and arcs (gene-to-gene relationships) are indicated on the magnified view. The thickness of the arcs and their color indicates the strength of positive or negative correlation between the interconnected genes. Some of the pathway arcs are labled: +p = phosphorylation; −p = dephosphorylation; +u = ubiquitination; +m = methylation; e = expression;c = compound; and b = binding and unlabeled connecting arc implies activation.

A biological system model of the host response to MAP infection was created from the merger of 14 overlapping pathways that were considered to be major players in the host immune tolerance as identified and described above. Figure 9 depicts our conceptual holistic model of the interplay between pathways. This figure illustrates only a portion of the complex interplay that may be occurring as MAP subverts and hi-jacks different host biological processes. The actual systems Bayesian network is comprised of 433 genes constructed from known biological relationships contained in the overlapping pathways and resulted in a very dense network model as illustrated in Figure 10. This system-level network was interrogated to identify genes and key regulatory points (hubs) that are purported to be governing the host response to MAP. Since the model is trained by the host-pathogen response data, the computational nature of the dynamic Bayesian networks permits interrogation of the model both computationally and visually to identify correlated relationships and candidate regulator hubs that are potential targets for immune and/or therapeutic intervention. Table 22 illustrates the interrogation of the model for highly correlated downstream gene relationships for the important regulatory gene *AKT3*. AKT3 was identified as a key mechanistic gene and a gene with high overlap with multiple pathways. Ultimately, the diversion of the host gene response to benefit the pathogen depends on the activation of different genes in a particular pathway. This system model enabled a broader examination of the interrelated pathway-host response that we could not have otherwise identified from traditional statistical analysis methods. Further interrogation of the system model led to the identification of several mechanistic genes that have high positive correlated relationships and influence on downstream genes that included: *LEPR->Ppara*, *LEP->Stk11*, *SOCS3->JAK1*, *SOCS3->Irs2*, *STAT1->CXCL10*, *STAT3-Prkag2*, *MAP3K8->MAP3K14*, *NFKB1->IFNA13*, *NFKB2->TNF*, *JAK1->PTPN11*, *IFNAR2->STAT1*, *MAP3K14->IKBKB*, *TNF->TNFRSF1A*, and *NFKBIA->NFKBIB*. Some of the more dominating negative correlated relationships included: *NFKB1->IL8*, *STAT3->POMC*, *NFKB1->SOCS3*, *NFKB1->IL12B*, *NFKB1->CXCL10*, and *LEPR->CAMKK2*. The identification of these relationships provides important insight into possible points of interaction between MAP and the host and novel points of intervention.

**Table 22:** Illustrative Interrogation of the Model for Mechanistic *AKT3* Gene Downstream Regulatory Effects on Multiple Pathways.

Start Gene	Start Gene Description	Normalized Correlation Weight	End Gene	End Gene Description	Relationship
AKT3	V-akt murine thymoma viral oncogene homolog3 (protein kinase B, gamma)	−0.425	NFκB1	Nuclear factor of kappa light polypeptide gene enhancer in B cells 1	Phosphorylation
NFκB1	Nuclear factor of kappa light polypeptide gene enhancer in B cells 1	−0.143	CXCL-10	Chemokine (C-X-C motif) ligand 10	Indirect
NFκB1	Nuclear factor of kappa light polypeptide gene enhancer in B cells 1	−0.18	IL-12B	Interleukin 12B (NK cell stimulator factor 2), Cytotoxic lymphocyte maturation factor 2	Indirect
NFκB1	Nuclear factor of kappa light polypeptide gene enhancer in B cells 1	−0.185	SOCS3	Suppressor of cytokine signaling 3	Expression
NFκB1	Nuclear factor of kappa light polypeptide gene enhancer in B cells 1	−0.123	IL-6	Interleukin 6	Indirect
SOCS3	Suppressor of Cytokine Signalling 3	0.389	JAK1	Janus Kinase 1 (a protein tyrosine kinase)	Inhibition
SOCS3	Suppressor of Cytokine Signalling 3	0.257	Irs2	AGENCOURT_7772254 NIH_MGC_92 cDNA clone IMAGE:6014829 5′	Inhibition
SOCS3	Suppressor of Cytokine Signalling 3	0.125	LEPR	Leptin Receptor	Inhibition
JAK1	Janus kinase 1 (a protein tyrosine kinase)	0.179	PTPN11	Protein tyrosine phosphate, non-receptor type 11	Phosphorylation
LEPR	Leptin Receptor	0.484	Ppara	Peroxisome proliferator activated receptor alpha	Activation
LEPR	Leptin Receptor	0.243	Stk11	Serine/threonine kinase 11	
LEPR	Leptin Receptor	0.159	Camkk1	Calcium/Calmodulin-dependent protein kinase kinase 1, alpha	
LEPR	Leptin Receptor	0.141	Camkk2	Calcium/Calmodulin-dependent protein kinase kinase 1, beta	Activation

## Conclusions

The temporal *in vivo* host global gene expression analysis of the MAP infected major target organ in the target animal species provides unique opportunities to systematically identify and define the complexities of major pathways influencing the pathogenesis of Johne's Disease, particularly during the early, intermediate and late phase responses in the first 12 hours post-infection. Our Bayesian analysis and modeling of host gene expression data considerably strengthen the hypothesis that MAP subverts the bovine host innate and adaptive immune responses toward immune tolerance. More specifically, we identified no less than ten major cellular pathways that were subverted to reduce host cellular uptake and phagocytosis of MAP one of which is supported by our *in vitro* RNAi silencing of the mechanistic *MAPK1* gene resulting in highly significant reduced invasion of MAP. Furthermore, our analyses disclosed that MAP compromised the host mucosal immune barrier by manipulating the major mechanistic genes of the junction (gap, tight, adherens), cell adhesion molecules – intergrin mediated pathways, and the trefoil factor initiated mucosal healing pathway, adding credibility that the MAP induced decreased trans-epithelial resistance as revealed in our *in vitro* model and likely has considerable *in vivo* significance. Finally, we created a robust biological system model of the bovine host response to MAP infection facilitating computational and visual interrogation of the model to identify several potential targets for intervention. We demonstrated that the systems biology approach not only facilitated observations of a holistic functional picture of early responses to MAP, but also uncovered new pathways reinforcing immune tolerance while identifying mechanistic pathways compromising the enteric mucosal immune barrier during colonization of Peyer's patch by MAP.

## Supporting Information

Figure S1 **Significantly Perturbed Pathways of All Phases.** This heat map figure shows all scored pathways meeting a 97.5% confidence threshold at any time point and contains the same pathways as listed in Tables 2, 3 and 4. Starting at the left hand column top pathway, the order of the pathways are from the highest activated pathway score (i.e., Parkinson's Disease highest score at t = 30 min.) to the most suppressed pathway score (i.e., Thiamine metabolism lowest score at t = 720 min.) in the bottom right column. In this figure, the pathway scores are shown as darker red gradients indicating higher activation scores (more up-regulated gene expression within the pathway gene set) while the darker green gradients indicating more suppressed pathway activity (more down-regulated gene expression). Grey is near a zero score and black is equal to a zero score.
(DOC)

Table S1 **Significantly Perturbed GO Categories for Early Phase Response.** List of significantly perturbed gene ontology categories of biological processes for the Early Phase time period (30 and 60 minutes post-infection). Scores are determined by Dynamic Bayesian Gene Group Activation technique as explained in the text.
(XLS)

Table S2 **Significantly Perturbed GO Categories for Intermediate Phase Response.** List of significantly perturbed gene ontology categories of biological processes for the Intermediate Phase time period (120, 240, and 480 minutes post-infection). Scores are determined by Dynamic Bayesian Gene Group Activation technique as explained in the main text.
(XLS)

Table S3 **Significantly Perturbed GO Categories for Late Phase Response.** List of significantly perturbed gene ontology categories of biological processes for the Late Phase time period (720 minutes post infection). Scores are determined by Dynamic Bayesian Gene Group Activation technique as explained in the main text.
(XLS)

Table S4 **Mechanistic Genes Identified by Dynamic Bayesian Modeling.** State of modulation is indicated by the “+" for up-regulation and “−" for down-regulation.
(XLS)

Table S5 **Mechanistic Gene Cross-Talk.** This Table provides list of mechanistic genes for 43 pathways involved in signaling and immune response. Several mechanistic genes are associated in multiple pathways that may be the source of cross-talk, and thus, have more significant influence governing the host immune tolerance to MAP. Of the 43 pathways analyzed, 36 pathways had at least one overlapping mechanistic gene. It was found that 141 mechanistic genes had overlaps within the 36 pathways examined.
(XLS)
