# Supplementary material for: Systems Biology Analysis of Gene Expression during In Vivo Mycobacterium avium paratuberculosis Enteric Colonization Reveals Role for Immune Tolerance
Source: PLoS One. 2012 Aug 17;7(8):e42127. doi: 10.1371/journal.pone.0042127 (PMC3422314; doi:10.1371/journal.pone.0042127)
Supplement: Figure S1 — Significantly Perturbed Pathways of All Phases. This heat map figure shows all scored pathways meeting a 97.5% confidence threshold at any time point and contains the same pathways as listed in Tables 2, 3 and 4. Starting at the left hand column top pathway, the order of the pathways are from the highest activated pathway score (i.e., Parkinson's Disease highest score at t = 30 min.) to the most suppressed pathway score (i.e., Thiamine metabolism lowest score at t = 720 min.) in the bottom right column. In this figure, the pathway scores are shown as darker red gradients indicating higher activation scores (more up-regulated gene expression within the pathway gene set) while the darker green gradients indicating more suppressed pathway activity (more down-regulated gene expression). Grey is near a zero score and black is equal to a zero score. (DOC) [file pone.0042127.s001.doc]

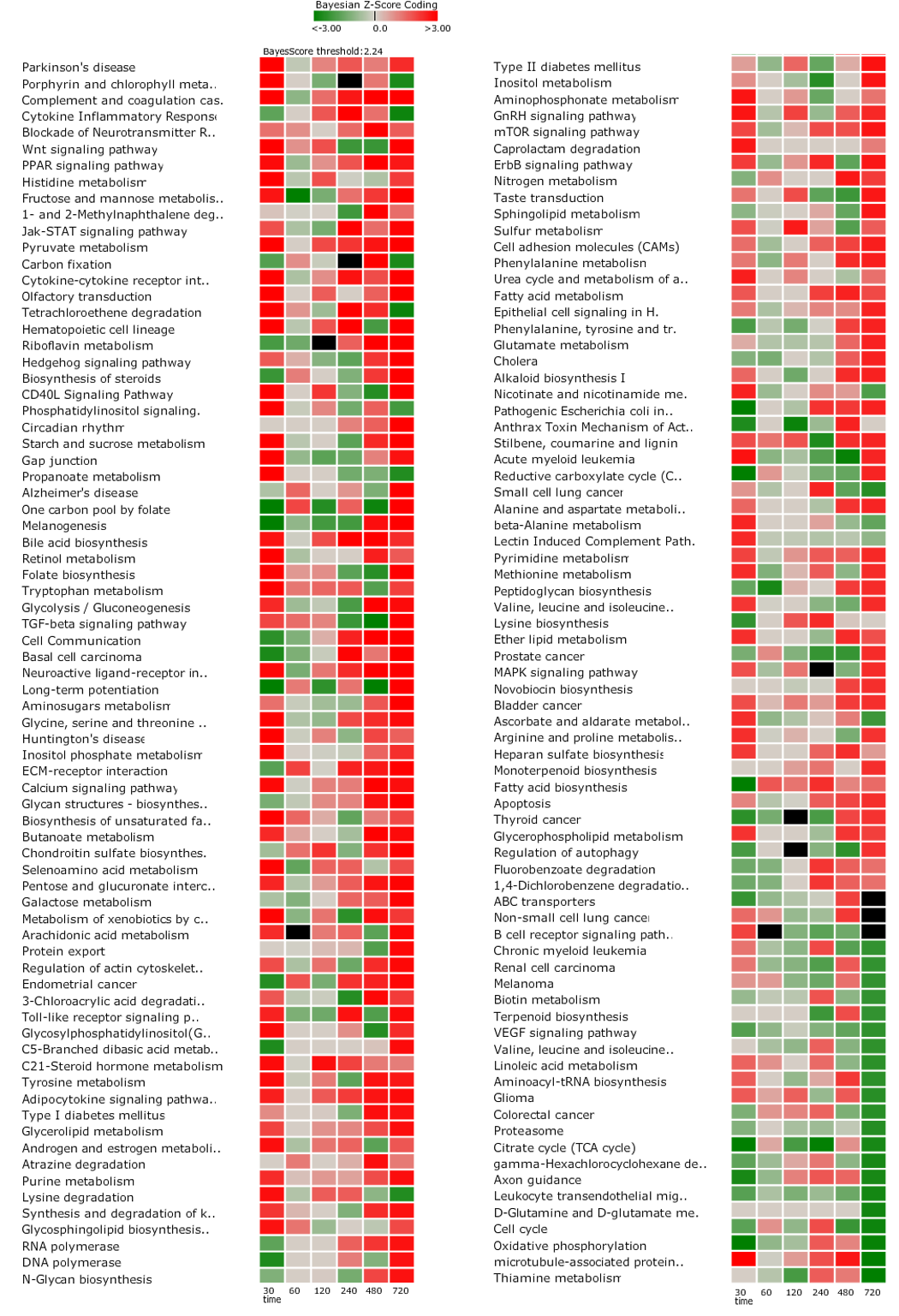


Figure S1. This heat map shows all scored pathways meeting a 97.5% confidence threshold at any time point and contains the same pathways as listed in Table 2 (a), (b), (c). Starting at the left hand column top pathway, the order of the pathways are from the highest activated pathway score (i.e., Parkinson’s Disease highest score at t=30 min.) to the most suppressed pathway score (i.e., Thiamine metabolism lowest score at t=720 min.) in the bottom right column. In this figure, the pathway scores are shown as darker red gradients indicating higher activation scores (more up-regulated gene expression within the pathway gene set) while the darker green gradients indicating more suppressed pathway activity (more down-regulated gene expression). Grey is near a zero score and black is equal to a zero score.
